# Increased tissue stiffness triggers contractile dysfunction and telomere shortening in dystrophic cardiomyocytes

**DOI:** 10.1016/j.stemcr.2021.04.018

**Published:** 2021-05-20

**Authors:** Alex C.Y. Chang, Gaspard Pardon, Andrew C.H. Chang, Haodi Wu, Sang-Ging Ong, Asuka Eguchi, Sara Ancel, Colin Holbrook, John Ramunas, Alexandre J.S. Ribeiro, Edward L. LaGory, Honghui Wang, Kassie Koleckar, Amato Giaccia, David L. Mack, Martin K. Childers, Chris Denning, John W. Day, Joseph C. Wu, Beth L. Pruitt, Helen M. Blau

**Affiliations:** 1 Department of Cardiology and Shanghai Institute of Precision Medicine, Ninth People's Hospital, Shanghai Jiao Tong University School of Medicine, A419, Bldg #2, 115 Jinzun Road, Pudong New District, Shanghai 200125, China; 2 Baxter Laboratory for Stem Cell Biology, Department of Microbiology and Immunology, Institute for Stem Cell Biology and Regenerative Medicine, Stanford University School of Medicine, Stanford, CCSR Room 4215, 269 Campus Drive, Stanford, CA 94305-5175, USA; 3 Division of Cardiovascular Medicine, Stanford University School of Medicine, Stanford, CA, USA; 4 Stanford Cardiovascular Institute, Stanford University School of Medicine, Stanford, CA, USA; 5 Departments of Bioengineering and Mechanical Engineering, Stanford University, School of Engineering and School of Medicine, Stanford, CA, USA; 6 Mechanical Engineering and Biomolecular Science and Engineering, University of California, Santa Barbara, CA, USA; 7 Division of Radiation and Cancer Biology, Department of Radiation Oncology, Stanford University, Stanford, CA, USA; 8 Department of Rehabilitation Medicine, Institute for Stem Cell and Regenerative Medicine, University of Washington, Seattle, WA, USA; 9 Division of Cancer & Stem Cells, Biodiscovery Institute, University of Nottingham, University Park NG7 2RD, UK; 10 Department of Neurology, Stanford University, Stanford, CA, USA

**Keywords:** dilated cardiomyopathy, telomere, hiPSC-CM, DMD, fibrosis

## Abstract

Duchenne muscular dystrophy (DMD) is a rare X-linked recessive disease that is associated with severe progressive muscle degeneration culminating in death due to cardiorespiratory failure. We previously observed an unexpected proliferation-independent telomere shortening in cardiomyocytes of a DMD mouse model. Here, we provide mechanistic insights using human induced pluripotent stem cell-derived cardiomyocytes (hiPSC-CMs). Using traction force microscopy, we show that DMD hiPSC-CMs exhibit deficits in force generation on fibrotic-like bioengineered hydrogels, aberrant calcium handling, and increased reactive oxygen species levels. Furthermore, we observed a progressive post-mitotic telomere shortening in DMD hiPSC-CMs coincident with downregulation of shelterin complex, telomere capping proteins, and activation of the p53 DNA damage response. This telomere shortening is blocked by blebbistatin, which inhibits contraction in DMD cardiomyocytes. Our studies underscore the role of fibrotic stiffening in the etiology of DMD cardiomyopathy. In addition, our data indicate that telomere shortening is progressive, contraction dependent, and mechanosensitive, and suggest points of therapeutic intervention.

## Highlights

• DMD hiPSC-CMs exhibit aberrant calcium handling and defective force generation
• DMD hiPSC-CMs undergo proliferation-independent telomere shortening
• Telomere shortening activates the p53 DNA damage pathway
• Telomere shortening in DMD hiPSC-CMs is contraction dependent

## Teaser

In this article, Chang, Blau, and colleagues show that Duchenne muscular dystrophy (DMD) iPSC-derived cardiomyocytes exhibit proliferation-independent telomere shortening, p53 activation and mitochondrial dysfunction.

## Introduction

Duchenne muscular dystrophy (DMD) is caused by >200 mutations in the gene encoding dystrophin, a protein that connects the cytoskeleton to the extracellular matrix, with a prevalence of 1:5,000 boys, DMD patients exhibit early and progressive skeletal muscle degeneration and weakness. However, patients succumb from dilated cardiomyopathy and respiratory failure (McNally et al., 2015), usually before the age of 30 years. In the heart, DMD manifests as electrocardiogram abnormalities, diastolic dysfunction, fibrosis, systolic dysfunction and fibrosis, which culminate in heart failure (Finsterer and Cripe, 2014). AAV-mediated gene therapy and gene-editing strategies show great promise in animal models (Amoasii et al., 2018; Chamberlain and Chamberlain, 2017). Yet, to date, DMD patients receive non-specific heart failure treatments, beta-blockers, or ACE inhibitors, largely due to a lack of understanding of the mechanisms underlying the cardiac failure.

We previously uncovered an unexpected association of dystrophin deficiency with short telomeres, the hexanucleotide TTAGGG repeats that cap and protect the ends of chromosomes. This stemmed from experiments directed at resolving the conundrum that the mdx^4cv^ mouse model, which lacks dystrophin as in DMD patients but has a normal lifespan, does not manifest the cardiac disease from which patients succumb. Strikingly, human telomeres (~10 kb) are significantly shorter than mouse telomeres (>40 kb). This led us to test our hypothesis that “humanizing” telomere lengths in the mdx^4cv^ mouse might reveal a disease phenotype with greater fidelity to human DMD. Indeed, we found that the dilated cardiomyopathy phenotype was faithfully recapitulated in mdx^4cv^/mTR^G2^ mice in which telomere lengths were shortened by breeding of the mdx^4cv^ mouse with the mouse TERC knockout (mTR), the RNA component of telomerase (Chang et al., 2016; Mourkioti et al., 2013). Moreover, cardiomyocytes in murine mdx^4cv^/mTR^G2^ and in DMD patient cardiac tissues exhibited ~50% reduction in telomere signal as measured by quantitative fluorescence *in situ* hybridization (Q-FISH) relative to mdx^4cv^/mTR^Het^ mouse controls or cardiomyocytes in age-matched human controls (Chang et al., 2016; Mourkioti et al., 2013). However, the mechanism by which this proliferation-independent telomere shortening is triggered remains unknown.

To gain insight into the molecular and cellular etiology of telomere shortening, here we model human DMD cardiomyopathy in culture and chart disease progression using cardiomyocytes differentiated from patient and isogenic control human induced pluripotent stem cells (hiPSC-CMs), a robust system for studying cardiac defects (Karakikes et al., 2015; Wu et al., 2019b). Importantly, we establish a bioengineered platform that allows for cardiomyocyte orientation with a 1:7 aspect ratio and mimics the stiffness of the DMD fibrotic heart. We show that DMD hiPSC-CMs exhibit aberrant calcium handling, contractile defects, and proliferation-independent telomere shortening. Furthermore, this telomere shortening is post-mitotic, progressive, and contraction dependent. By culturing hiPSC-CMs on a bioengineered platform that mimics the stiffness of healthy and fibrotic myocardium, we provide evidence that contractile dysfunction is exacerbated on a stiff myocardium characteristic of the DMD heart.

## Results

### Duchenne hiPSC-CMs recapitulate pathogenic features of dilated cardiomyopathy

**Figure 1:**
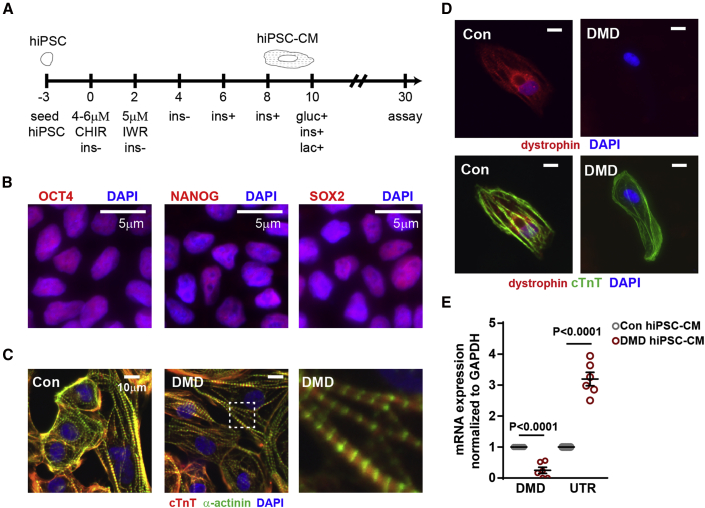
Generation of DMD hiPSC-CMs (A) hiPSC differentiation protocol for the generation of hiPSC-CMs. CHIR, CHIR-99021; IWR, IWR-1; ins, insulin; gluc, glucose; lac, lactate. hiPSCs were differentiated in medium supplemented with B27 minus insulin (ins). On day 6, the medium was supplemented with B27, including insulin. On day 10, hiPSC-CMs were cultured in medium without glucose supplemented with B27 and lactate. (B) Representative micrograph in which hiPSCs were stained with pluripotent stem cell markers OCT4, NANOG, and SOX2. (C) Representative micrograph in which hiPSC-CMs were stained with cardiac troponin T, α-actinin, and DAPI. (D) Representative micrograph in which healthy and DMD hiPSC-CMs were stained with dystrophin, cardiac troponin T, and DAPI. (E) Endogenous dystrophin (DMD) and utrophin (UTR) expression levels were determined by qRT-PCR in hiPSC-CMs (n = 6 independent experiments). Data shown as the mean ± SEM. Student's t test was used to calculate significance.

To study the molecular underpinnings of telomere shortening in DMD hiPSC-CMs, we differentiated six DMD and six control hiPSC lines into beating cardiomyocytes using well-established protocols (Burridge et al., 2015) (Table S1; Figure 1A). Our studies utilized hiPSC lines from four different laboratories derived from disparate cell types, including two isogenic pairs in which dystrophin mutations were corrected by CRISPR-Cas9 (Con1 and 2 matching DMD1 and 2) (Dick et al., 2013), and one isogenic pair in which a deletion of the first six exons was introduced into healthy cells by CRISPR-Cas9 (Con3 matching DMD3) (Guan et al., 2014; Pioner et al., 2020), three non-familial healthy controls (Con4, 5, and 6), and three DMD lines (DMD4, 5, and 6) (Table S1). Pluripotency markers for hiPSC were evaluated by immunofluorescence staining for pluripotency markers OCT4, NANOG, and SOX2 (Figure 1B), and beating cardiomyocytes were determined to express hallmark proteins, cardiac troponin (cTnT), and α-actinin (Figure 1C). The presence or absence of dystrophin was confirmed by immunofluorescence (Figure 1D) as well as by qRT-PCR (Figure 1E).

**Figure 2:**
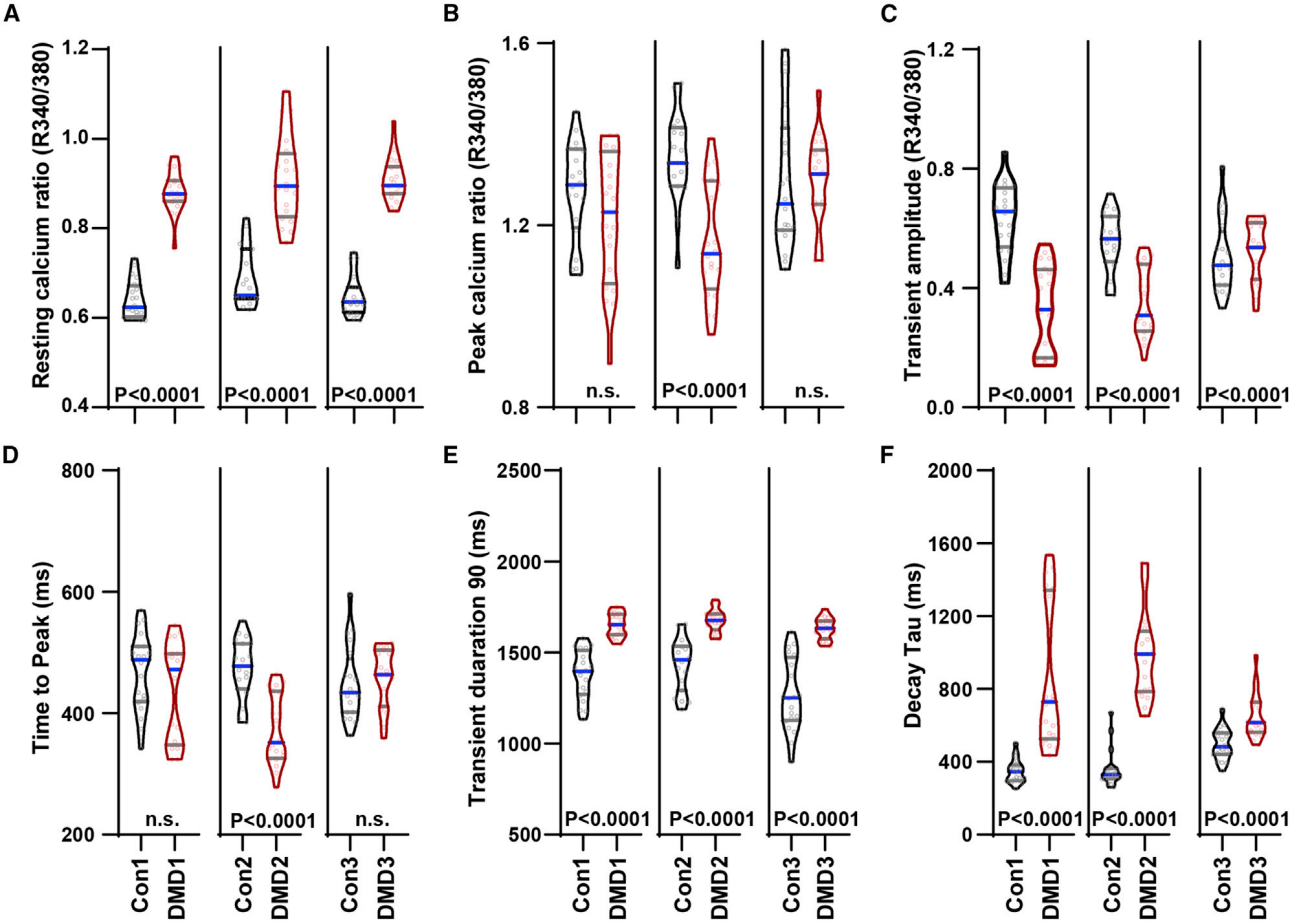
DMD hiPSC-CMs exhibit aberrant calcium-handling properties Using ratiometric-based Fura2 calcium imaging, (A) resting calcium ratio, (B) peak calcium ratio, (C) transient amplitude, (D) time to peak, (E) transient duration 90, and (F) decay tau per isogenic hiPSC pairs were plotted (isogenic pairs only; n = 3 independent experiments, 19–20 cells analyzed). Data are shown as violin plots where blue median and gray quartiles are shown. Student's t test was used to calculate significance.

To evaluate basal calcium levels, ratiometric Fura2 measurements under 1 Hz pacing conditions were used, as described previously (Wu et al., 2019a). We observed increased varied resting calcium ratio (Figure 2A), decreased trend in peak calcium ratio (Figure 2B), decreased transient amplitude (Figure 2C), no difference in time to peak except Con2/DMD2 (Figure 2D), increased transient duration 90 (Figure 2E), and increased decay tau (Figure 2F). Similar trends were observed for spontaneous calcium transients using the Fluo-4 method as described previously (Wu et al., 2015). Compared with controls, DMD hiPSC-CMs exhibited aberrant calcium transients (Figure S1A) with decreased transient amplitude, with the exception of Con2/DMD2 (Figure S1B), delayed time to peak (Figure S1C), prolonged TD50 (Figure S1D) but no difference in decay tau (Figure S1E), similar to previously characterized calcium transients in DMD (Guan et al., 2014; Pioner et al., 2020) and other genetic dilated cardiomyopathy hiPSC-CMs (Sun et al., 2012; Wu et al., 2015). Together, our results confirm that DMD hiPSC-CMs exhibited aberrant calcium handling using two different calcium transient imaging methods, while showing good line to line consistency.

### DMD hiPSC-CMs exhibit dysfunctional contractile force generation when subjected to increased mechanical load

**Figure 3:**
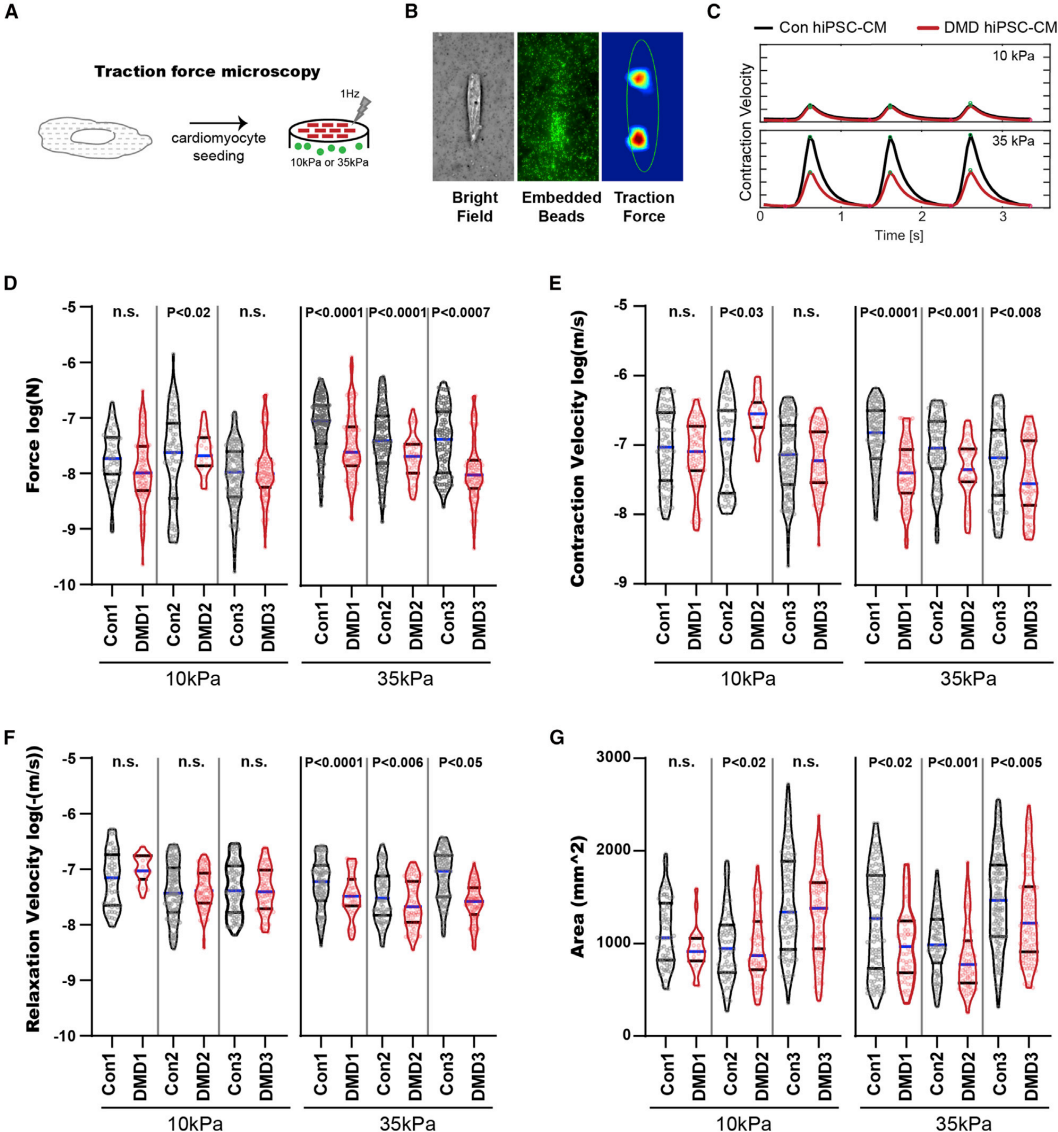
DMD hiPSC-CMs exhibit contractile dysfunction under fibrotic microenvironment challenge (A) Contractile assessment of DMD hiPSC-CMs using traction force microscopy where hiPSC-CMs were seeded onto micropatterned tunable hydrogel devices. (B and C) (B) Representative micrographs of contraction force generated by a single hiPSC-CM on 10 or 35 kPa hydrogel (bright field, GFP fluorescent beads, Fourier traction force cytometry) and (C) representative contraction cycles are shown. (D–G) (D) Force, (E) contraction velocity, (F) relaxation velocity, and (G) cell area of single Con and DMD hiPSC-CMs subjected to 10 and 35 kPa hydrogels were measured (n = 3 independent experiments, 19–143 cells analyzed). Data are shown as violin plots where blue median and gray quartiles are shown. Student's t test was used to calculate significance.

To measure DMD hiPSC-CM contractile force, we developed a traction force microscopy platform that mimics the stiffness of the heart tissue before (10 kPa) and after the onset of fibrosis (35 kPa) (Engler et al., 2008) using a tunable hydrogel (Figure 3A) (Ribeiro et al., 2015). Single hiPSC-CMs were configured by micropatterns to assume a physiological 7:1 length:width aspect ratio, characteristic of adult cardiomyocytes and previously shown to promote hiPSC-CM sarcomere alignment and maturation (Ribeiro et al., 2015). Our traction force microscopy platform captures the force generated by single cardiomyocytes as a function of hydrogel deformation measured by tracking the displacement of embedded fluorescent beads (Figure 3B), and traction stress is then reconstructed using Fourier transform traction cytometry. The contractile parameters, including force and velocity, were measured in several contraction cycles in DMD and healthy hiPSC-CMs cultured on 10 and 35 kPa hydrogel devices (Figure 3C, Video S1. Bright field of control iPSC-CMs on biopatterned hydrogel, Video S2. Fluorescent channel of control iPSC-CMs on biopatterned hydrogel, Video S3. Traction heatmap of control iPSC-CMs on biopatterned hydrogel, Video S4. Bright field of DMD iPSC-CMs on biopatterned hydrogel, Video S5. Fluorescent channel of DMD iPSC-CMs on biopatterned hydrogel, Video S6. Traction heatmap of DMD iPSC-CMs on biopatterned hydrogel).

Video S1. Bright field of control iPSC-CMs on biopatterned hydrogel

Video S2. Fluorescent channel of control iPSC-CMs on biopatterned hydrogel

Video S3. Traction heatmap of control iPSC-CMs on biopatterned hydrogel

Video S4. Bright field of DMD iPSC-CMs on biopatterned hydrogel

Video S5. Fluorescent channel of DMD iPSC-CMs on biopatterned hydrogel

Video S6. Traction heatmap of DMD iPSC-CMs on biopatterned hydrogel

A significant decrease in force generation was observed in DMD hiPSC-CMs compared with controls on 35 kPa, but not on 10 kPa hydrogels (Figure 3D). DMD hiPSC-CMs on 35 kPa hydrogel devices also exhibited a decrease in contraction velocity (Figure 3E), relaxation velocity (Figure 3F), and cell surface area (Figure 3G), which is consistent with our calcium-handling measurements. The fact that contractile deficiency was only apparent in DMD hiPSC-CMs cultured on 35 kPa substrates suggests that DMD cardiomyocytes are unable to compensate for dystrophin deficiency once the heart tissues undergo fibrotic stiffening, a hallmark of DMD cardiomyopathy.

Under basal conditions, DMD hiPSC-CMs exhibit increased reactive oxygen species (ROS) (Figure S2A), but no difference in mitochondrial respiration was detected using the Seahorse bioanalyzer (Figures S2B and S2C). However, it is known that cardiomyocytes switch to glycolytic metabolism in heart failure conditions (Bertero and Maack, 2018). Under nutrient starvation (Figure S2D), glucose only (Figure S2E), pyruvate only (Figure S2F), or palmitate only (Figure S2G) conditions, DMD hiPSC-CMs exhibit a significant decrease in basal oxygen consumption rate and maximal oxygen consumption rate. Together, these data provide strong evidence that DMD hiPSC-CMs exhibit metabolic maladaptation and contractile dysfunction similar to that seen in DMD heart failure progression.

### Telomere shortening in the absence of cell division

**Figure 4:**
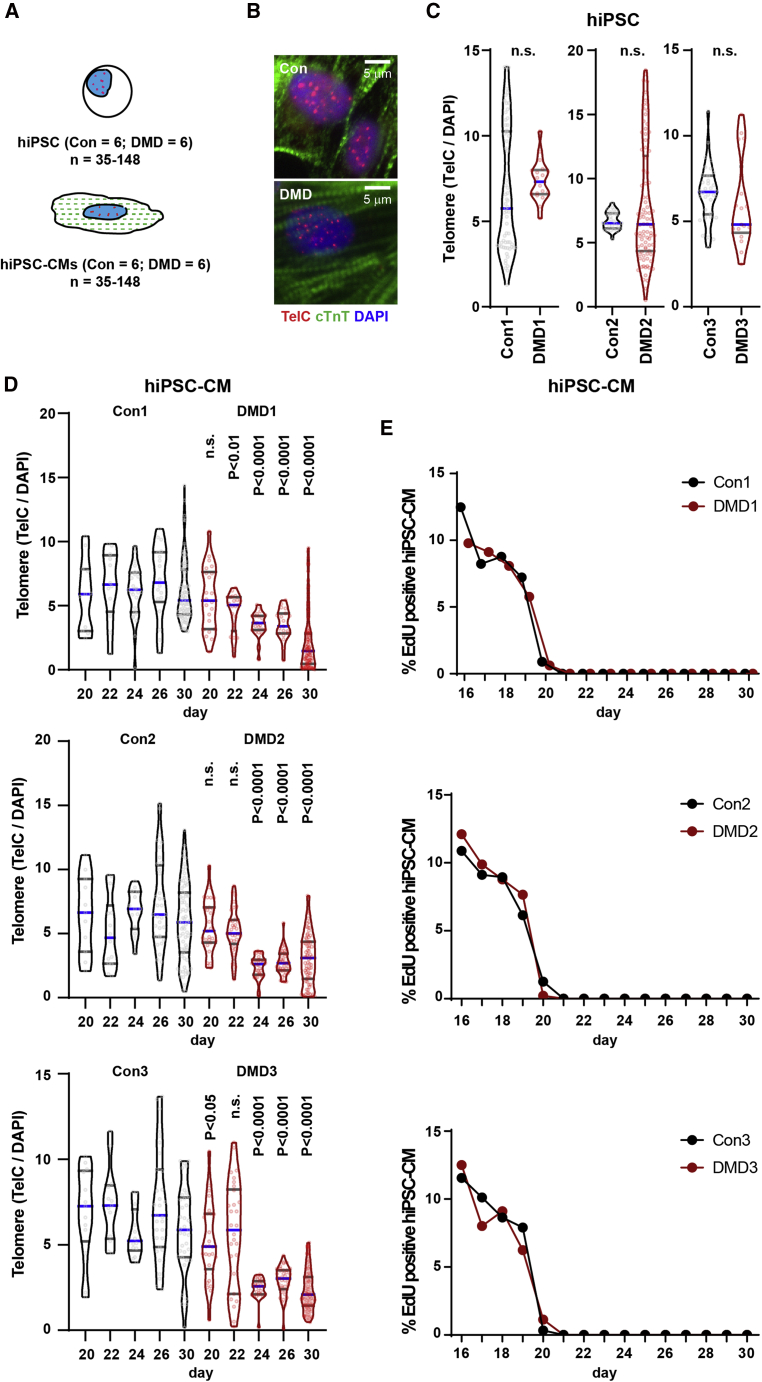
DMD hiPSC-CMs exhibit telomere shortening and DNA damage response (A) Telomere length (TelC) was quantified by immunofluorescence staining (Q-FISH) relative to nuclear DAPI staining for hiPSC and hiPSC-CMs. (B) Representative micrographs of hiPSC-CMs stained with cardiac troponin T, TelC and DAPI are shown. (C) Quantification of hiPSC telomere using Q-FISH (n = 3 independent experiments, 17–86 cells analyzed). (D) Telomere Q-FISH reveals progressive telomere loss in DMD hiPSC-CMs between days 20 and 30 (n = 3 independent experiments, 11–179 cells analyzed). (E) hiPSC-CMs were devoid of EdU between days 20 and 30. Data are shown as violin plots where blue median and gray quartiles are shown. Student's t test was used to calculate significance.

We sought to determine if DMD hiPSC-CMs would exhibit telomere shortening similar to that seen in cardiomyocytes of human DMD cardiac tissues (Chang et al., 2018) and mdx^4cv^/mTR^G2^ cardiac tissues (Chang et al., 2016). To measure telomeres, we used Q-FISH and measured the telomere signal of proliferative hiPSCs and differentiated hiPSC-CMs over a time course of differentiation (Figures 4A and 4B). Telomere signals of proliferating hiPSCs did not differ statistically for healthy controls and DMD hiPSCs, or for the three isogenic pairs (Figures 4C and S3A).

Telomere loss is typically a “passive” process that accompanies incomplete replication at each cell division in the course of aging (Blackburn et al., 2015; López-Otín et al., 2013). The hiPSC-CM platform allowed us to test if cardiomyocytes can undergo telomere shortening in the absence of DNA replication and cell division. This is of particular interest since cardiomyocytes are largely post-mitotic in mice and humans during postnatal development (Bergmann et al., 2015; Porrello et al., 2011). To address this possibility, we assessed telomere lengths in conjunction with DNA replication measured by EdU (5-ethynyl-2’-deoxyuridine) incorporation (24 h pulse) over a time course of hiPSC-CM differentiation, where day 0 is defined as the first day of differentiation and each day thereafter is the time point post induction of differentiation (Figures 4D, 4E, and S4A). In agreement with our DMD mouse (mdx^4cv^/mTR^G2^) and DMD patient results (Chang et al., 2016; Mourkioti et al., 2013), we observed progressive telomere shortening, reaching 50% telomere reduction by day 30 in DMD hiPSC-CMs compared with controls (Figures 4D and S4A). EdU labeling revealed that proliferation is still occurring at day 16 but ceases in hiPSC-CMs between days 20 and 30 (Figures 4E, S4B, and S4C). Interestingly, it was during this post-mitotic period that we observed a progressive telomere loss in DMD hiPSC-CMs compared with controls culminating in a ~50% reduction by day 30 (Figures 4D and S4A).

**Figure 5:**
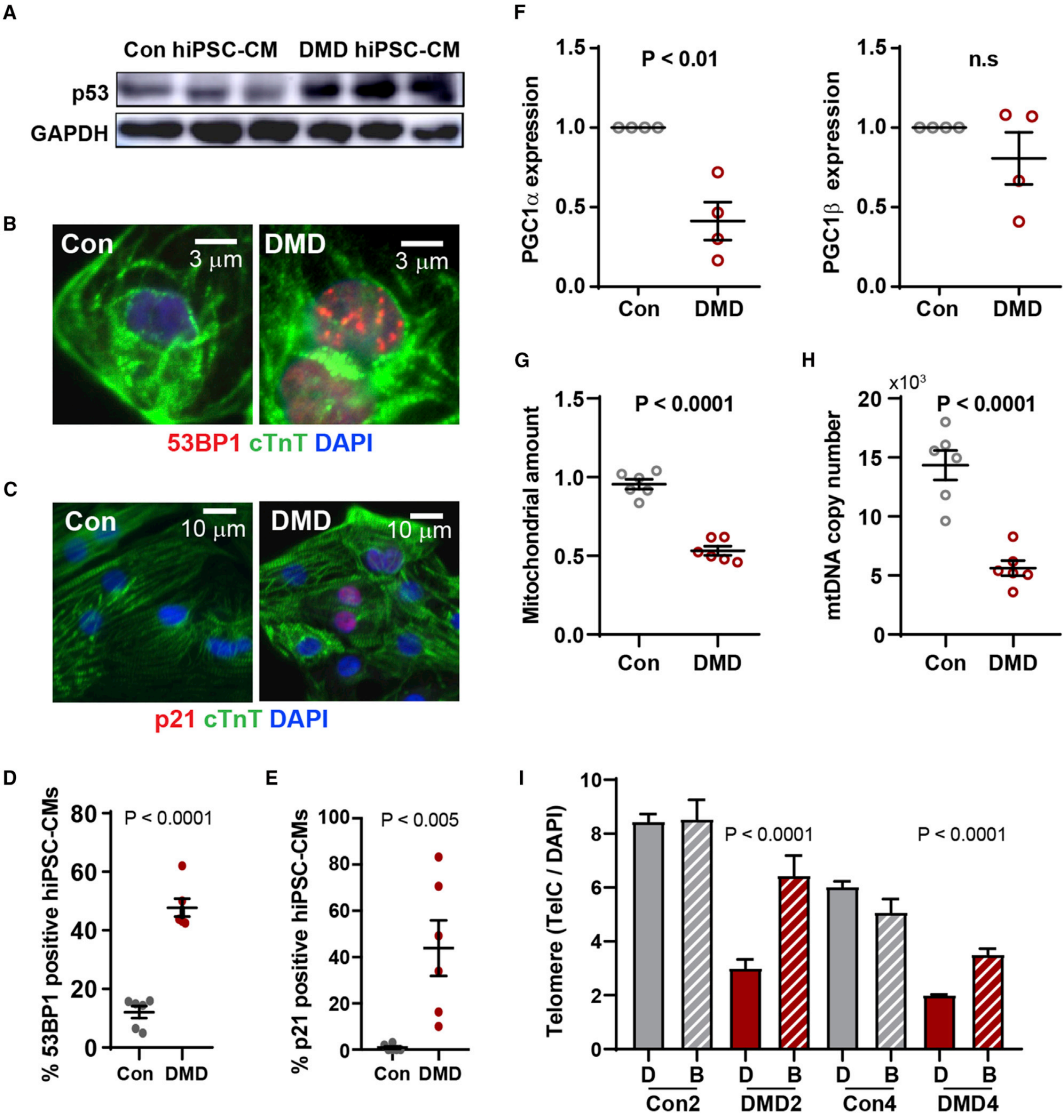
DMD hiPSC-CMs exhibit p53 upregulation (A–E) Representative micrographs of (A) p53 activation by immunoblotting (n = 3 independent experiments), (B) DNA damage 53BP1 foci by immunofluorescence (n = 6 independent experiments), and (C) p21 (n = 6 independent experiments) in cardiac troponin T+ hiPSC-CMs are shown and (D and E) quantified, respectively. (F) Reduced expression levels of PGC-1α, master regulator of mitochondrial biogenesis, determined by qRT-PCR (n = 6 independent experiments). (G and H) (G) Mitochondria amount (n = 6 independent experiments) and (H) mitochondrial copy number (n = 6 independent experiments) in hiPSC-CMs were assessed by MitoTracker Green and qRT-PCR using mitochondrial gene (Nd2) to nuclear DNA (Nrf1) primers, respectively. Data represent mean ± SEM. Student's t was test used for statistical analysis. (I) Telomere loss prevented when contraction of DMD hiPSC-CMs was inhibited between days 20 and 30 using blebbistatin was quantified (n = 3 independent experiments, 35–1,834 cells analyzed). Data represent mean ± SEM. Student's t test was used for statistical analysis.

We postulated that an early molecular trigger of telomere shortening could be telomere deprotection via loss of the shelterin proteins that cap the telomeres. To address this possibility, we isolated cardiomyocytes on day 20, at the onset of proliferation-independent telomere shortening, using flow cytometric enrichment for hiPSC-CMs based on mitochondrial membrane potential assessed by TMRM staining, as described previously (Chang et al., 2018). qRT-PCR revealed decreased transcript levels of telomere repeat binding proteins (*TRF1* and *TRF2*) and shelterin complex proteins (*RAP1*, *TIN2*, and *POT1*) in DMD relative to control hiPSC-CMs (Figure S3D). To determine whether shortened telomeres led to the activation of the DNA damage response, we assessed the levels of p53 (Figure 5A), the number of the p53 binding protein 1 (53BP1) foci per single cell (Figures 5B and 5D), and p21 levels (Figures 5C and 5E). We found that p53 protein was upregulated in DMD hiPSC-CMs (Figure 5A) and that the incidence of 53BP1-positive hiPSC-CMs increased from 10% to 50% in control and DMD, respectively (Figures 5D and S4E). However, we did not detect a significant difference in day 30 DMD hiPSC-CMs compared with controls when we scored cells with telomere-induced foci defined as more than three 53BP1 foci colocalized with telomere probe (Figure S3E) (Takai et al., 2003). Furthermore, this 53BP1 accumulation is correlated with the expression of p53 downstream target p21 protein (Figures 5C and 5E). Activation of the DNA damage response, as indicated by elevated 53BP1, induces p53-mediated repression of PGC-1α, which in turn impedes mitochondrial biogenesis (Figures 5F–5H). Moreover, we observed an increase in apoptotic markers caspase-3 (Figure S3F), cleaved PARP (Figure S3G), and accumulation of β-galactosidase signal in DMD hiPSC-CMs compared with healthy controls (Figure S3H). Interestingly, when we blocked DMD hiPSC-CM contraction using blebbistatin daily starting at day 20, with a dose that locks the myosin heads in a low affinity state, thus preventing actin binding (Skwarek-Maruszewska et al., 2009), telomere shortening was abrogated, as determined by measurements on day 30 (Figure 5I). Here, our data show shelterin gene downregulation, with concomitant telomere shortening independent of cell division due to aberrant contraction, elicits a p53-dependent DNA damage response.

## Discussion

We provide evidence for progressive telomere shortening via a previously unrecognized replication-independent mechanisms in hiPSC-derived cardiomyocytes that harbor mutations in the structural protein, dystrophin. This is remarkable, as beyond early postnatal development, the heart is a relatively non-proliferative organ (Bergmann et al., 2015; Porrello et al., 2011). We demonstrated previously that humanizing the telomere lengths unmasks the dilated cardiomyopathy phenotype in the mouse model of DMD (mdx^4cv^) (Chang et al., 2016; Mourkioti et al., 2013) as well as bicuspid valve phenotype in Notch haploinsufficient (Notch^+/−^) mice (Theodoris et al., 2017). Telomere attrition occurs “passively” in proliferative systems due to the end-replication problem during cell division (Arnoult and Karlseder, 2015; Sfeir and de Lange, 2012). Using the DMD hiPSC-disease model, we were able to chart the dynamics of telomere shortening and show that it occurs independent of proliferation in DMD hiPSC-CMs, corroborating and extending snapshots of the pathogenic process previously seen in murine and human cardiac tissues (Chang et al., 2016, 2018; Mourkioti et al., 2013).

The DMD hiPSC-CM model and the use of traction force microscopy on stiff hydrogels reported here faithfully recapitulates features of cardiomyopathy in DMD patients: aberrant calcium handling and contractile dysfunction (Finsterer and Cripe, 2014), increased ROS production (Chang et al., 2016), and decreased contractile force (Finsterer and Stollberger, 2003; Sasaki et al., 1998). Moreover, our results are in agreement with dilated cardiomyopathy phenotypes seen in other genetic etiologies, e.g., troponin T deficiency (Sun et al., 2012; Wu et al., 2015). Importantly, we provide insights into the mechanism by which an increased stiffness following the development of fibrosis in the DMD myocardium at later stages compromises the contractile function of DMD hiPSC-CMs. Our results suggest that cardiomyocytes can compensate for the lack of dystrophin in the healthy heart (10 kPa), but not the stiff, fibrotic heart (35 kPa), which could explain why DMD patients become increasingly susceptible to arrhythmia and heart failure as they age. Our characterization of the contractile response to microenvironments of different rigidities highlights the development of an acute mechanical dysfunction in DMD cardiomyocytes in conjunction with the progression of fibrosis in the heart.

The robustness of our findings is underscored by the fact that we used hiPSC lines from six DMD and six controls, generated in four different labs from four diverse starting cell types, including two isogenic lines comprised of CRISPR-corrected DMD mutations and one isogenic line that was a CRISPR-created mutant. In agreement with these reports, irrespective of hiPSC origin, DMD or control, we detected similar markedly long telomere lengths at the outset in hiPSCs. Strikingly, upon differentiation into cardiomyocytes, DMD hiPSC-CMs exhibited a dramatic reduction in telomere lengths relative to controls, affording a cell model to study and monitor telomere shortening in DMD cardiomyocytes. Our observations corroborate the marked telomere reduction that we and others previously detected in cardiomyocytes of genetic cardiomyopathic patients (Chang et al., 2018) or cardiac tissues from patients who succumbed to heart failure (Oh et al., 2003; Terai et al., 2013).

A molecular trigger for telomere shortening appears to be the loss of the shelterin complexes (Sfeir and de Lange, 2012). In accordance, we find that binding of shelterin complexes to the telomeres in DMD cardiomyocytes is reduced at the onset of proliferation-independent telomere shortening, possibly due to the stress of contraction in the absence of dystrophin. Based on the observed ~30% increase in ROS production in DMD hiPSC-CMs and previous findings by others showing high ROS can drive telomere oxidation and disrupt shelterin binding (Opresko et al., 2005), we speculate that direct oxidation of uncapped telomere repeats may also play a role in the observed telomere shortening (von Zglinicki et al., 1995). In agreement, we showed that shelterin expression is reduced in DMD hiPSC-CMs, and others have shown that this occurs in human cardiomyopathy tissue (Oh et al., 2003). However, whether loss of shelterin expression is the cause or consequence of telomere deprotection in DMD cardiomyocytes remains to be determined. Furthermore, ROS production is driven, in part, by contractile activity in DMD cardiomyocytes (Prosser et al., 2011). Our finding that blebbistatin treatment impedes telomere shortening suggests that contractile function plays a crucial role. Whether this is due to ROS-mediated telomere deprotection or disruption of sarcomere organization remains to be elucidated. Nonetheless, these data provide support for our hypothesis that contraction in conjunction with the genetic absence of a key structural contractile protein plays a causal role in telomere shortening, as reported here in the iPSC-CM disease model and previously in the mouse model of DMD (Chang and Blau, 2018). In parallel, loss of structural integrity may induce transcriptional changes, as demonstrated in laminopathic cardiomyocytes (Cheedipudi et al., 2019). Downstream sequelae include induction of a DNA damage response evident by accumulation of 53BP1, p53, and p21 proteins, mitochondrial demise, and metabolic failure in DMD hiPSC-CMs, in agreement with mTR^G4^ mouse models of aging (Sahin et al., 2011) and the DMD mouse model with “humanized” telomeres (mdx^4cv^/mTR^G2^) (Chang et al., 2016).

The acquisition of a “senescence-like state” by DMD hiPSC-CMs is suggested by the expression of 53BP1, p53, p21, and β-galactosidase, as recently described in other non-dividing systems (Gorgoulis et al., 2019). Although the animal models support the importance of telomere length in cardiomyopathy pathogenesis (Chang et al., 2016; Sahin et al., 2011), a prolonged DNA damage response may disrupt other pathways important for cell survival, such as the PGC-1α repression of mitochondrial biogenesis. Whether mitochondrial dysfunction plays a role in the pathogenesis remains to be elucidated (Correia-Melo et al., 2016). We hypothesize that telomere shortening is at play in other stressed post-mitotic differentiated cell types, such as neurons harboring mutations (Ain et al., 2018) or acquired defects (Aguado et al., 2019). Together, our findings provide evidence showing that telomere shortening occurs independent of proliferation and is potentially driven by contraction-induced ROS in dystrophic cardiomyocytes. Our results suggest a novel therapeutic target, telomere maintenance, to ameliorate or halt the progression of DMD cardiomyopathy regardless of mutational status.

## Experimental procedures

### Statistics

Statistical differences between patient hiPSC and hiPSC-CM groups were analyzed using one-way analysis of variance by comparing the mean of each group with the mean of every other group, followed by Holm-Sidak’s multiple comparison test (non-isogenic groups) or by two-tailed unpaired Student’s t tests (isogenic pairs). Image capture and quantification analyses were performed in a double-blinded fashion to avoid bias. All data are shown as the mean ± SEM. Significant differences were determined as p < 0.05. For a detailed summary of all statistical calculations please see Data S1.

### hiPSC culture and differentiation into hiPSC-CMs

All protocols using hiPSC were reviewed and approved by the Stanford Stem Cell Research Oversight committee (#602) as well as the Ethics Review committee at Ninth People's Hospital, Shanghai Jiao Tong University School of Medicine (2018-207-K32). hiPSCs were grown on Matrigel-coated (Corning, 356231) plates using Nutristem medium. The medium was changed daily, and cells were passaged every 4 days using Accutase (Sigma, A6964) and seeded in 1:8 dilution with addition of 5 μM Y-27632 2HCl (Selleck Chem, S1049). hiPSCs were grown to 70%–90% confluence and subsequently differentiated into beating cardiomyocytes, as demonstrated previously (Chang et al., 2018). Beating hiPSC-CMs were purified against non-cardiomyocytes and matured by culturing in glucose-free conditions using RPMI-1640 medium without glucose with B27 supplement and 5 mM lactate (Life Technologies) until day 30 (Hinson et al., 2015; Ni et al., 2021; Tohyama et al., 2013).

### Blebbistatin treatment

Beating day 20 hiPSC-CMs were treated with 5 μm blebbistatin (Abcam, ab120425) to lock the myosin heads in a low affinity state for actin to prevent contraction (Skwarek-Maruszewska et al., 2009) as per the manufacturer's recommendation. Blebbistatin and DMSO were first mixed with RPMI-1640 medium without glucose with B27 supplement and 5 mM lactate (Life Technologies), the medium was changed daily until day 30. hiPSC-CMs were then fixed for downstream telomere quantification.

### Fluorescence-activated cell sorting purification of hiPSC-CMs

Fluorescence-activated cell sorting (FACS) purification was carried out for qRT-PCR data. Using a described previously protocol (Chang et al., 2018; Tohyama et al., 2013), hiPSC-CMs were purified using FACS. Cell cultures were dissociated using Accutase and stained with 50 nM tetramethylrhodamine methyl ester perchlorate (TMRM). Cells were gated for side scatter and forward scatter to avoid debris and doublets and TMRM+ (cardiomyocytes) were isolated for downstream characterization.

### Immunofluorescence and Q-FISH

hiPSC-CMs and hiPSCs were fixed with 4% paraformaldehyde in PBS for 5 min at room temperature and subsequently maintained in PBS at 4°C. For the Q-FISH time course, day 18 hiPSC-CMs were seeded onto Matrigel-coated 8-chamber slides and fixed at various days as indicated. Telomere Q-FISH was performed as described previously using TelC-Cy3 PNA probe (CCCTAACCCTAACCCTAA) (PNA Bio, F1002) (Chang et al., 2016; Mourkioti et al., 2013). Samples were blocked with staining buffer (20% fetal bovine serum/0.1% Triton X-100/PBS) and stained with rabbit anti-Oct4 (1:400, Abcam, ab19857), rabbit anti-Sox2 (1:400, Abcam, ab92494), rabbit anti-Nanog (1:400, Abcam, ab80892), rabbit anti-53BP1 (1:400, Bethyl, IHC-00001), p21 Waf1/Cip1 (Cell Signaling Technology, 2947S), rabbit anti-cleaved PARP1 (1:400, Abcam, ab32064), rabbit anti-cleaved caspase-3 Asp175 (1:400, CST, no. 9661), rabbit α-actinin (1:400, Thermo Scientific, 42-1400), rabbit anti-dystrophin (1:100, Abcam, ab15277), and/or mouse anti-cardiac troponin T antibody (1:500, Abcam, ab74275 or ab8295) for 2 h at room temperature or overnight at 4°C in staining buffer, washed, and stained with goat anti-mouse or anti-rabbit Alexa 488, 594, or 647 (1:400, Abcam) for 1 h, washed and counterstained with 1 μg mL^−1^ DAPI in PBS for 5 min, washed with dH_2_O, air dried, and mounted with ProLong Gold Antifade (Life Technologies). To assay cell proliferation, hiPSC-CMs were pulsed with 24 h EdU and proliferation was assessed using the Click-iT EdU Alexa Fluor 488 Imaging Kit (Thermo Fisher Scientific). Images were captured on a Nikon Spinning Disk Confocal microscope using a PLAN APO 40x objective. Telomere signal intensity for cardiomyocytes (troponin T+) was determined as PNA signal (TelC probe) in each nucleus normalized to the DAPI intensity of that nucleus using ImageJ plugin Telometer, as described previously (Chang et al., 2016; Meeker et al, 2002, 2004; Mourkioti et al., 2013).

### Immunoblotting

Immunoblotting was performed as described previously (Chang et al., 2016). In brief, 30 μg of total RIPA protein lysate was separated on a NuPAGE Bis-Tris 4%–12% gel under MOPS condition (Thermo Scientific), transferred to a PVDF membrane (Sigma-Aldrich), and immunoblotted using mouse anti-p53 (1:2000, ProteinTech, 10442-1-AP) and rabbit anti-GAPDH (1:3,000, CST, no. 5174) antibodies followed by goat anti-mouse (1:10,000, A16072, Sigma-Aldrich) and goat anti-rabbit HRP (1:10,000, G-21234, Sigma-Aldrich).

### qRT-PCR

RNAs were extracted using the Direct-zol RNA MiniPrep Plus with Zymo-Spin IIICG Columns (Zymo Research, R2070), the quantity and quality of RNA was determined using a NanoDrop 2000 spectrophotometer (Thermo Fisher Scientific), and cDNA was reverse transcribed using a High-Capacity cDNA Reverse Transcription Kit (Life Sciences/ABI, 4374966). Total DNA were extracted using a DNeasy Blood & Tissue Kit (QIAGEN, 69506) and the quantity and quality of DNA were determined using a NanoDrop 2000 spectrophotometer. mRNA expression levels were detected using TaqMan probes for the following genes: DMD (Hs00758098), UTR (Hs01126016), TRF1 (Hs00819517_mH), TRF2 (Hs01030567_m1), TPP1 (Hs00166099_m1), TIN2 (Hs01554309_g1), RAP1 (Hs00430292_m1), POT1 (Hs00209984_m1), PGC1α (Hs01016719_m1), PGC1β (Hs00991677_m1), and GAPDH (Hs02758991_g1). Fold enrichment was calculated using 2ˆ-ΔΔCT normalized to GAPDH, then to healthy controls. Mitochondrial copy number assay was performed as described previously (Evdokimovsky et al., 2011) using the following TaqMan probes: NRF1 (Hs04926189_cn), ND2 (Hs02596874_g1).

Calcium transient, Seahorse bioanalyzer and ROS measurements, and traction force microscopy.

## Author contributions

A.C.Y.C. and H.M.B. conceived and designed the research. G.P., A.J.S.R., and B.L.P. conceived and designed traction force microscopy. A.C.Y.C., G.P., A.C.H.C., K.K., H. Wu, S.-G.O., A.E., S.A., C.H., J.R., A.J.S.R., E.L., and H. Wang performed the research. A.C.Y.C., G.P., A.C.H.C., K.K., H. Wu., S.A., and H.M.B. analyzed the data. All authors contributed to the writing of the manuscript.

## Declaration of Interests

The authors declare no competing interests.

## Supplemental information

Document S1. Supplemental experimental procedures and Figures S1–S3

Document S2. Data S1

Document S3. Article plus supplemental information
