# Supplementary material for: Increased tissue stiffness triggers contractile dysfunction and telomere shortening in dystrophic cardiomyocytes
Source: Stem Cell Reports. 2021 May 20;16(9):2169–81. doi: 10.1016/j.stemcr.2021.04.018 (PMC8452491; doi:10.1016/j.stemcr.2021.04.018)
Supplement: Document S1. Supplemental experimental procedures and Figures S1–S3 [file mmc1.pdf]

Stem Cell Reports, Volume *16*

Supplemental Information

Increased tissue stiffness triggers contractile dysfunction and telomere shortening in dystrophic cardiomyocytes

Alex C.Y. Chang, Gaspard Pardon, Andrew C.H. Chang, Haodi Wu, Sang-Ging Ong, Asuka Eguchi, Sara Ancel, Colin Holbrook, John Ramunas, Alexandre J.S. Ribeiro, Edward L. LaGory, Honghui Wang, Kassie Koleckar, Amato Giaccia, David L. Mack, Martin K. Childers, Chris Denning, John W. Day, Joseph C. Wu, Beth L. Pruitt, and Helen M. Blau

Online Data Supplements

Supplemental Experimental Procedures

Calcium transient

For calcium transient measurement, hiPSC-CMs were disassociated by Accutase and seeded in Matrigel-coated 8-well LAB-TEK® II cover glass imaging chambers (Thermo Fisher) at a density of 20,000 cells per well. Ratiometric calcium, Fura2, imaging was performed as previously described (H. Wu et al., 2019). After recovery, cells were loaded with 5 μM Fluo-4 AM in Tyrode's solution (140 mM NaCl, 5.4 mM KCl, 1 mM MgCl2, 10 mM glucose, 1.8 mM CaCl2, and 10 mM HEPES, pH 7.4 adjusted with NaOH at RT) for 10 min in 37°C incubator. Cells were then washed with pre-warmed Tyrode's solution for 3 times. Spontaneous calcium transient was sampled by confocal microscope (Carl Zeiss, LSM 510 Meta, Göttingen, Germany) with a 63X oil immersed objective (Plan-Apochromat 63x/1.40 Oil DIC M27). Signal was captured using line-scanning mode (512 pixels X 1920 lines). Custom-made IDL (interactive digital language) script was used for data analysis. Transient amplitude was expressed as $\Delta F/F_0$.

Seahorse bioanalyzer and ROS measurements

HiPSC-CMs were seeded at 30,000 per well in Matrigel-coated Seahorse plates and basal culturing conditions were performed as previously described (Guan et al., 2014). For nutrient challenge, hiPSC-CMs were cultured in RPMI-1640 B27$^+$ glucose$^-$ lactate$^+$ three days prior to assay. HiPSC-CMs were switched to Seahorse

unbuffered RPMI-1640 supplemented with B27$^+$ glucose$^-$ lactate$^+$ prior to the assay run. The bioenergetic responses of cells were measured with the Seahorse Bioscience XF96 flux Analyzer following instruction in the XF cell Mito Stress Test Kit User Guider. All oxygen consumption rate (OCR) measurements were acquired at 5-minute intervals with 1-minute mixing step between. Three baseline measurements were acquired followed by injection of oligomycin to a final concentration of 2.5 μM. After three measurements in presence of oligomycin, FCCP was injected to a final concentration of 1 μM and three measurements were recorded. Lastly, rotenone and antimycin A were injected to reach a final concentration of 2 μM followed by three measurements. For nutrient challenge assays, cardiomyocytes were subjected to four metabolic substrate conditions: basal assay medium (Seahorse assay medium), D-glucose (5 μM), Palmitate (167 μM), or Pyruvate (5 μM) and loaded with oligomycin (5 μM), FCCP (5 μM), rotenone and antimycin A (1.69 μM). OCR was normalized to live cell count using a PrestoBlue dye according to manufacturer's protocol. PrestoBlue (A-13261, Life Tech), Mitotracker Green (M7514, Thermo Scientific) and CellROX deep red (C10422, Thermo Scientific) were measured and quantified on a TECAN Pro1000 machine (Stanford High-Throughput Bioscience Center).

Traction Force Microscopy

Micropatterned hydrogel devices of 10 kPa and 35 kPa stiffness were manufactured 1-day prior to passaging and seeding of day 25 hiPSC-CMs. Imaging and data acquisition were consistently performed 4-5 days after seeding the cells on

the devices. HiPSC-CM derived from Isogenic pairs were always measured consecutively on the same day. HiPSC-CM derived from each cell line were measured in differentiation triplicates.

To generate micropatterned hydrogel devices, polydimethylsiloxane stamps (PDMS) (PDMS-182, Sylgard, mixed at 1:10 ratio) were prepared by replica molding of SU8-silicon master mold produced in house using photolithography (Ribeiro et al., 2015). A master mold containing several 1 cm^2 arrays of micropatterns of 1:7 (width:length) aspect ratio and 118 x 17 μm (~2000 μm^2) area was prepared on 4" single-side polished silicon wafer using UV lithography in 10 µm-thick SU8-2010 resin (MicroChem, USA) using a collimated light source (OAI) (130 mJ/cm^2) and a high-resolution film photomask (FineLineImaging, USA), chemical development with SU-8 developer followed by hard-bake on a 180°C hotplate for 10 min. PDMS was mixed using a Thinky™ mixer, poured on the molds, degassed under vacuum for 1 h and cured in a 70°C oven for > 1 h. After carefully separating the solidified PDMS from the SU8-master mold, individual patterned stamps of were cut using a razor blade. Stamps were cleaned using a rotary shaker in a sequence of 5 min 1:1 H_2O:Ethanol followed by 5 min pure Ethanol. Stamps were subsequently dried with a gentle nitrogen stream. Stamps were cooled in a 4°C refrigerator before incubation at 4°C with 150 µl of Matrigel (Corning™ Matrigel™ hESC-Qualified Matrix, Catalog #: 08-774-552) dissolved 1:10 in Leibovitz's L-15 Medium (Thermo Fisher, Catalog #: 11415064) for a minimum of 12 hours. The stamps were then softly rinsed once with 150 µl of 4°C L-

15 medium to remove excess protein, the solution was then aspirated, and the stamps were carefully dried using a gentle nitrogen stream.

For microprinting and pattern transfer, glass cover slips of 18 mm diameter were cleaned using a rotary shaker in a sequence of 3min in Acetone followed by 3 min in Isopropanol. After drying with nitrogen, the coverslips were plasma treated for 20 s at 80 W in an oxygen plasma asher reactor (Branson IPC/Novellus). The protein patterns were then stamped onto the coverslips by carefully placing a PDMS stamp onto each freshly plasma activated glass coverslip. A glass slide was used as a carrier for the stamps and 50 g weights were placed on top of the stack for 2 min to maintain an even and constant pressure. The weights were then removed, and after 3 min the coverslips were removed from the stamps.

22mm-square coverslips were used as carrier substrates for the hydrogels and were functionalized by silanization to covalently bind the polyacrylamide gel to the glass. The coverslips were first cleaned and plasma activated, as above, and subsequently incubated with 0.3% 3-(trimethoxysilyl)propyl methacrylate (Sigma Aldrich, Catalog #: 440159) in 1:20 acetic acid glacial:ethanol (Sigma Aldrich, Catalogue #: 695092 and 459836) for 5 min. The excess solution was then aspirated, and the coverslips were rinsed using ethanol, dried with nitrogen and placed in a vacuum for a minimum of 30 min prior to usage.

Hydrogel devices were prepared by polymerization of freshly prepared and degassed polyacrylamide solution sandwiched between the transfer-substrate coverslip and the carrier substrates. Solutions of Acrylamide (Sigma Aldrich, Catalog

#: A9099) (0.5 g/ml), N,N′-Methylenebis(acrylamide) (Sigma Aldrich, Catalog # 146072) (0.025 g/ml), ammonium persulfate (APS) (Sigma) (0.1% w/v), N,N,N′,N′-tetramethylethylenediamine (TEMED)(Sigma Aldrich) (0.1% v/v), HEPES (Life Technologies) (35 mM), fluorescent microbeads (green/red fluorescent latex microsphere, diameter 0.5 μm, ThermoFischer, Catalogue #: F8813) and Milli-Q water were prepared and used throughout this study to maintain consistency. The two stiffnesses used in this study, 10 and 35 kPa, were obtained by mixing the prepolymer at 1% bisacrylamide to acrylamide ratio and 10% and 15% polymer to liquid-phase ratio, respectively. Fluorescence beads were sonicated for 10 min and pre-mixed 15% (v/v) in HEPES and added to obtain ~2% beads stock (~$6x10^9$ microbeads/mL) in the final gel solutions.

The hydrogel components were first mixed and degassed for 30 min in the absence of APS and TEMED. Immediately prior to making the gels, the APS and TEMED were added using a manual 2- and 10 μl-pipette, rapidly mixed with a 1 ml pipette, before dispensing a volume of 50 μl gel precursor on each square coverglass with a 200 μl pipette. The round coverslip containing the protein micropattern was then immediately placed on top of the gel precursor. The gel was allowed to polymerize for at least 30 min thereby transferring the micropatterns onto the solidifying hydrogel. Once polymerization was complete, the hydrogel devices were transferred into 6-well cell-culture dishes and incubated at room temperature overnight in Phosphate Buffer Saline (PBS) with 1% PenStrep to allow for swelling of the hydrogels. 15 min prior to seeding hiPSC-CMs, the top round coverglass used to transfer the protein

micropatterns was removed from the top of the hydrogel surface to expose the protein micropatterns using precision forceps. The PBS solution was aspirated, and the devices were pre-conditioned in a cell culture incubator at 37°C until seeding of the cells.

HiPSC-CMs were passaged onto micropatterned polyacrylamide devices by using 4:1 Accutase:TrypLE™ for dissociation and RPMI-1640 B27$^+$ glucose$^-$ lactate$^+$ medium with 5 μM ROCK inhibitor and 10% Knock-out Serum (KSR) for resuspension. After counting, cells were deposited onto the 18 mm diameter hydrogels at a density of ~30,000 cells/cm^2 in a final 150 µl volume at a concentration of 200,000 cell/ml. After 30 min incubation, additional 2 ml of medium was added. After overnight incubation, the medium was replaced with fresh RPMI-1640 B27$^+$ glucose$^-$ lactate$^+$ medium and replaced every 2 days thereafter.

Supplemental References

Guan, X., Mack, D.L., Moreno, C.M., Strande, J.L., Mathieu, J., Shi, Y., Markert, C.D., Wang, Z., Liu, G., Lawlor, M.W., Moorefield, E.C., Jones, T.N., Fugate, J.A., Furth, M.E., Murry, C.E., Ruohola-Baker, H., Zhang, Y., Santana, L.F., Childers, M.K., 2014. Dystrophin-deficient cardiomyocytes derived from human urine: new biologic reagents for drug discovery. Stem Cell Res 12, 467–480. doi:10.1016/j.scr.2013.12.004

Wu, H., Yang, H., Rhee, J.-W., Zhang, J.Z., Lam, C.K., Sallam, K., Chang, A.C.Y., Ma, N., Lee, J., Zhang, H., Blau, H.M., Bers, D.M., Wu, J.C., 2019. Modelling diastolic dysfunction in induced pluripotent stem cell-derived cardiomyocytes from hypertrophic cardiomyopathy patients. Eur. Heart J. 40, 3685–3695. doi:10.1093/eurheartj/ehz326

Supplemental Table S1. Summary of Control and DMD hiPSC lines used.

**Patient hiPSC lines**						
**Classification**	**hiPSC lines**	**Mutation**	**Age**	**Tissue**	**Gender**	**Source**
Healthy	Con#1	Isogenic control of DMD #1	10	Skin	male	University of Nottingham
Healthy	Con#2	Isogenic control of DMD #2	8	Skin	male	University of Nottingham
Healthy	Con#3	Isogenic control of DMD #3	16	Urine mesenchymal	male	University of Washington
Healthy	Con#4	Healthy control	45	PBMC	male	Stanford
Healthy	Con#5	Healthy control	62	T-Cells	male	Harvard
Healthy	Con#6	Healthy control	48	T-Cells	female	Harvard
DMD	DMD#1	DMD (c.10171C>T)	10	Skin	male	University of Nottingham
DMD	DMD#2	DMD (c.4918_4919delACinsTG)	8	Skin	male	University of Nottingham
DMD	DMD#3	DMD (c.19delG)	16	Urine mesenchymal	male	University of Washington
DMD	DMD#4	DMD (c.3638_3650del)	6	PBMC	male	Stanford
DMD	DMD#5	DMD (c.6599 C>G)	12	PBMC	male	Stanford
DMD	DMD#6	DMD (c.9204_9207del)	9	PBMC	male	Stanford

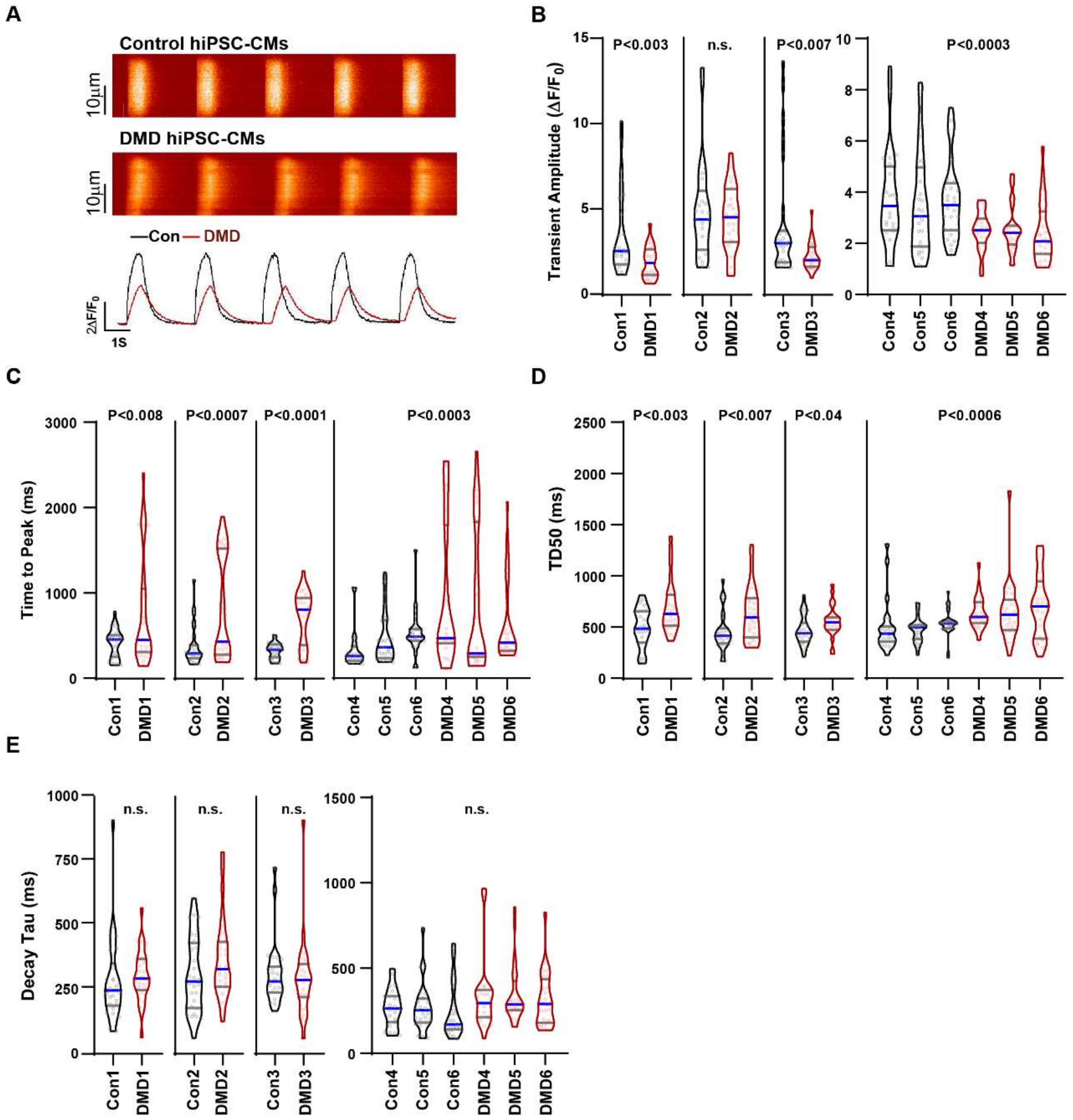

Figure S1. Evaluation of spontaneous calcium handling in control and DMD hiPSC-CMs. (**A**) Representative fluorescent based Fluo-4 calcium imaging recording traces in Con and DMD iPSC-CMs, (**B**) transient amplitude, (**C**) time to peak, (**D**) TD50, and (**E**) decay tau are plotted (n = 3 independent experiments, 24-28 cells analyzed). Data are shown as violin plots where blue median and gray quartiles are shown. Student's t-test used for Con/DMD 1-3. One-way ANOVA with Holm-Sidak's multiple comparison test used for Con/DMD 4-6.

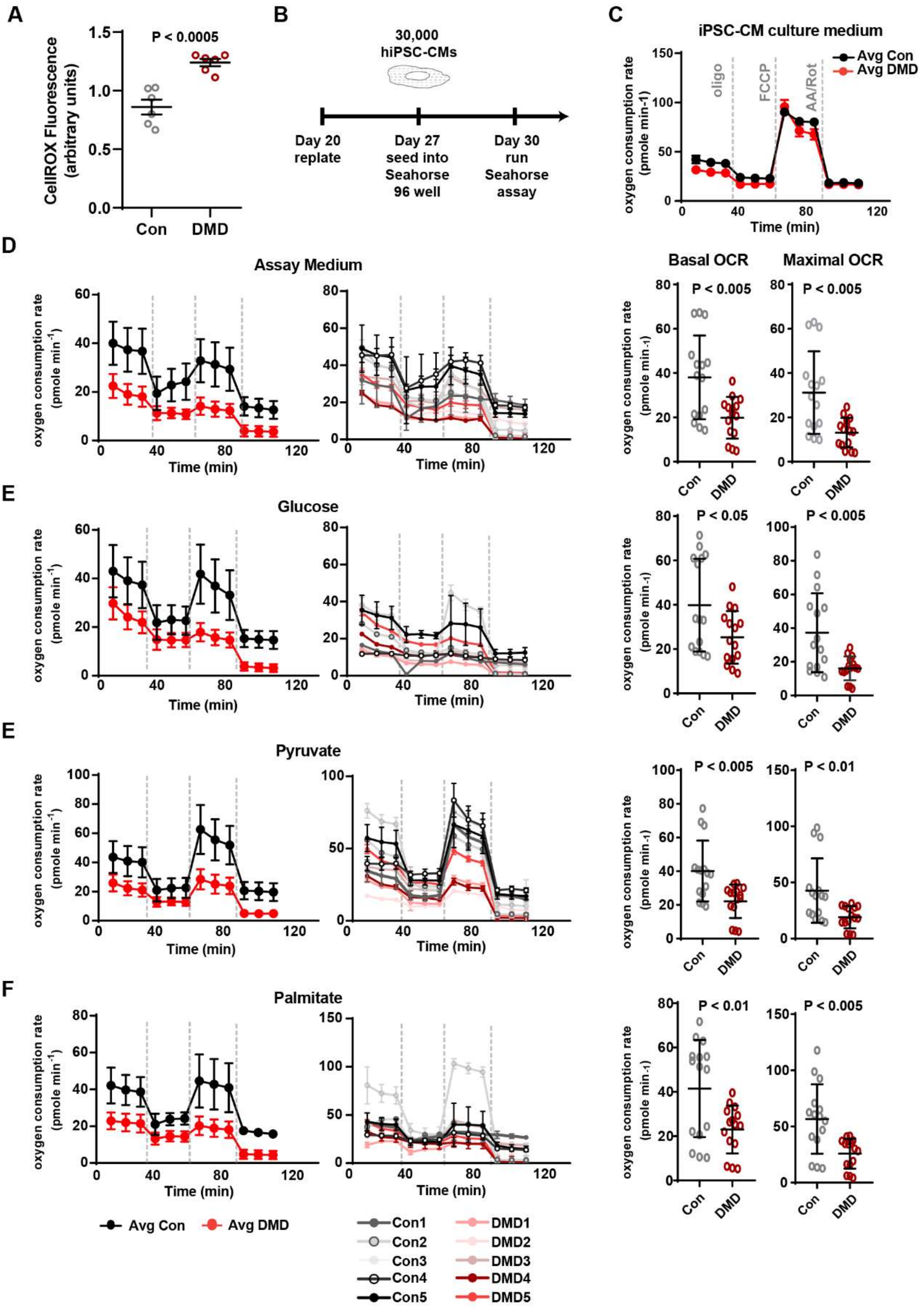

Figure S2. **DMD hiPSC-CMs exhibit mitochondrial dysfunction when subjected to nutrient challenge.** (**A**) Intracellular reactive oxygen species were measured in Con and DMD hiPSC-CMs (n = 6 independent experiments). (**B**) Real-time oxygen

consumption rate (OCR) were evaluated for control and DMD hiPSC-CMs under (**C**) normal culture conditions, (**D**) assay medium only, (**E**) glucose only, (**F**) pyruvate only, and (**G**) palmitate only conditions (n = 5 independent experiments). Data are represented as mean ± SEM. Student's t-test used for statistical analysis.

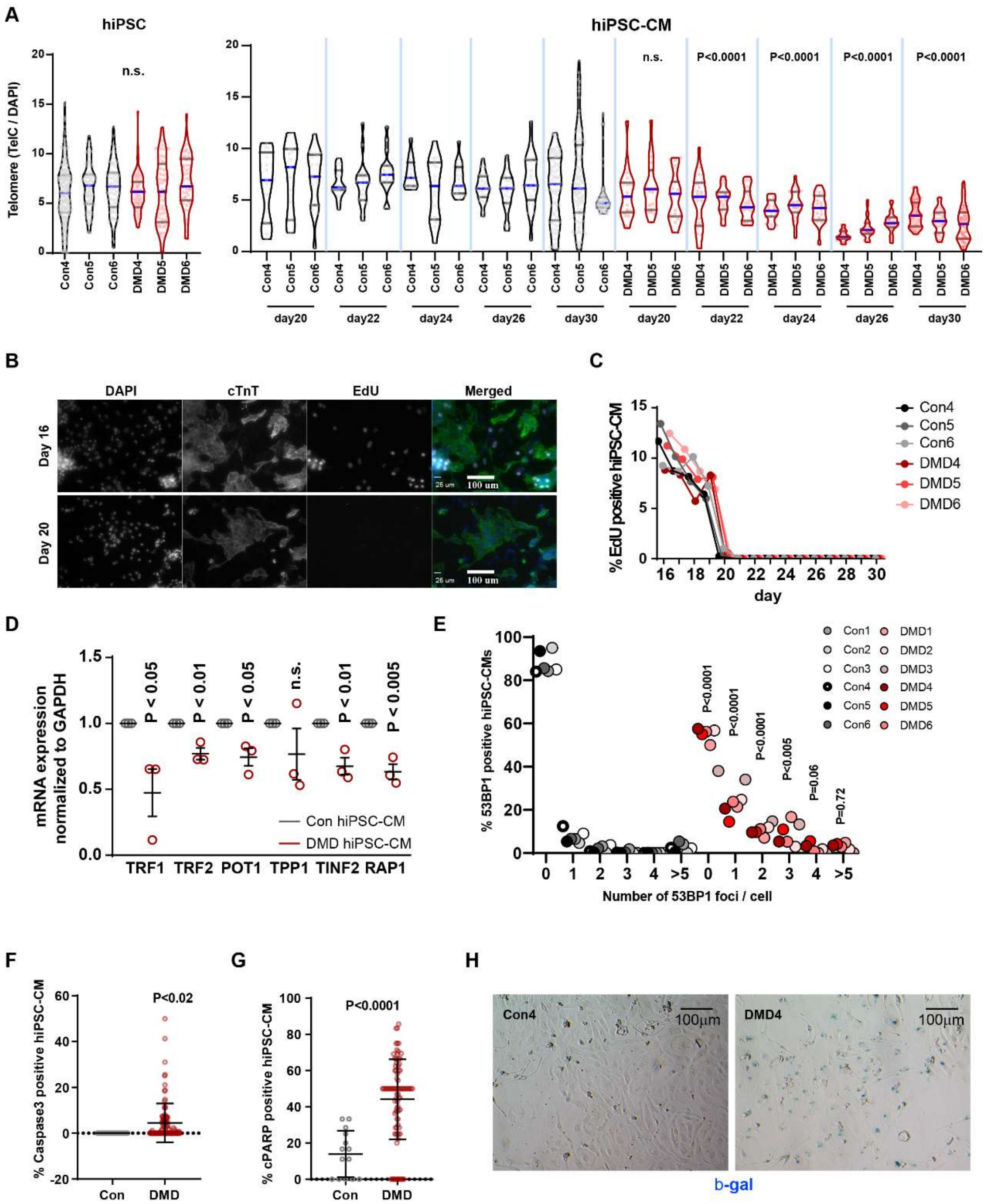

Supplemental Figure S3. (**A**)Quantification of hiPSC (n = 56-175 independent experiments) and hiPSC-CMs (n = 3 independent experiments, 13-108 cells analyzed) between days 20 to 30 of Con/DMD 4-6 lines. Data are shown as violin plots where blue median and gray quartiles are shown. One-way ANOVA with Holm-Sidak's multiple comparison test used for Con/DMD 4-6. (**B**) HiPSC-CM proliferation status evaluated by microscopy of EdU staining (24 hr pulses) and (**C**)

quantified for Con/DMD 4-6 lines. (**D**) Endogenous shelterin expression levels were determined by RT-qPCR in TMRM-purified day 20 hiPSC-CMs (n = 3 independent experiments). (**E**) Quantification of 53BP1 foci by immunofluorescence staining for Con and DMD hiPSC-CMs (n = 6 independent experiments). Data represent as mean ± SEM. Student's t-test used for statistical analysis. Quantification of (**F**) caspase 3 (n = 3 independent experiments, 23-101 cells analyzed) and (**G**) cleaved PARP (n = 3 independent experiments, 14-88 cells analyzed) by immunofluorescence staining for Con and DMD hiPSC-CMs. Data represent as mean ± SEM. Student's t-test used for statistical analysis. (**H**) Representative brightfield image of Control and DMD hiPSC-CMs stained for β-galactosidase.

Supplemental Videos:

Video S1. Brightfield of Control iPSC-CM on biopatterned hydrogel.

Video S2. Fluorescent channel of Control iPSC-CM on biopatterned hydrogel.

Video S3. Traction heatmap of Control iPSC-CM on biopatterned hydrogel.

Video S4. Brightfield of DMD iPSC-CM on biopatterned hydrogel.

Video S5. Fluorescent channel of DMD iPSC-CM on biopatterned hydrogel.

Video S6. Traction heatmap of DMD iPSC-CM on biopatterned hydrogel.
