# Supplementary material for: Increased tissue stiffness triggers contractile dysfunction and telomere shortening in dystrophic cardiomyocytes
Source: Stem Cell Reports. 2021 May 20;16(9):2169–81. doi: 10.1016/j.stemcr.2021.04.018 (PMC8452491; doi:10.1016/j.stemcr.2021.04.018)
Supplement: Document S3. Article plus supplemental information [file mmc9.pdf]

Stem Cell Reports
Article
OPEN ACCESS

Increased tissue stiffness triggers contractile dysfunction and telomere shortening in dystrophic cardiomyocytes

Alex C.Y. Chang,[1,2,3,4,*] Gaspard Pardon,[2,4,5,6] Andrew C.H. Chang,[2,3] Haodi Wu,[4] Sang-Ging Ong,[4] Asuka Eguchi,[2] Sara Ancel,[2] Colin Holbrook,[2] John Ramunas,[2] Alexandre J.S. Ribeiro,[4,5] Edward L. LaGory,[7] Honghui Wang,[1] Kassie Koleckar,[2] Amato Giaccia,[7] David L. Mack,[8] Martin K. Childers,[8] Chris Denning,[9] John W. Day,[10] Joseph C. Wu,[4] Beth L. Pruitt,[4,5,6] and Helen M. Blau[2,4,*]

[1]Department of Cardiology and Shanghai Institute of Precision Medicine, Ninth People's Hospital, Shanghai Jiao Tong University School of Medicine, A419, Bldg #2, 115 Jinzun Road, Pudong New District, Shanghai 200125, China
[2]Baxter Laboratory for Stem Cell Biology, Department of Microbiology and Immunology, Institute for Stem Cell Biology and Regenerative Medicine, Stanford University School of Medicine, Stanford, CCSR Room 4215, 269 Campus Drive, Stanford, CA 94305-5175, USA
[3]Division of Cardiovascular Medicine, Stanford University School of Medicine, Stanford, CA, USA
[4]Stanford Cardiovascular Institute, Stanford University School of Medicine, Stanford, CA, USA
[5]Departments of Bioengineering and Mechanical Engineering, Stanford University, School of Engineering and School of Medicine, Stanford, CA, USA
[6]Mechanical Engineering and Biomolecular Science and Engineering, University of California, Santa Barbara, CA, USA
[7]Division of Radiation and Cancer Biology, Department of Radiation Oncology, Stanford University, Stanford, CA, USA
[8]Department of Rehabilitation Medicine, Institute for Stem Cell and Regenerative Medicine, University of Washington, Seattle, WA, USA
[9]Division of Cancer & Stem Cells, Biodiscovery Institute, University of Nottingham, University Park NG7 2RD, UK
[10]Department of Neurology, Stanford University, Stanford, CA, USA
*Correspondence: alexchang@shsmu.edu.cn (A.C.Y.C.), hblau@stanford.edu (H.M.B.)
https://doi.org/10.1016/j.stemcr.2021.04.018

SUMMARY

Duchenne muscular dystrophy (DMD) is a rare X-linked recessive disease that is associated with severe progressive muscle degeneration culminating in death due to cardiorespiratory failure. We previously observed an unexpected proliferation-independent telomere shortening in cardiomyocytes of a DMD mouse model. Here, we provide mechanistic insights using human induced pluripotent stem cell-derived cardiomyocytes (hiPSC-CMs). Using traction force microscopy, we show that DMD hiPSC-CMs exhibit deficits in force generation on fibrotic-like bioengineered hydrogels, aberrant calcium handling, and increased reactive oxygen species levels. Furthermore, we observed a progressive post-mitotic telomere shortening in DMD hiPSC-CMs coincident with downregulation of shelterin complex, telomere capping proteins, and activation of the p53 DNA damage response. This telomere shortening is blocked by blebbistatin, which inhibits contraction in DMD cardiomyocytes. Our studies underscore the role of fibrotic stiffening in the etiology of DMD cardiomyopathy. In addition, our data indicate that telomere shortening is progressive, contraction dependent, and mechanosensitive, and suggest points of therapeutic intervention.

INTRODUCTION

Duchenne muscular dystrophy (DMD) is caused by >200 mutations in the gene encoding dystrophin, a protein that connects the cytoskeleton to the extracellular matrix, with a prevalence of 1:5,000 boys, DMD patients exhibit early and progressive skeletal muscle degeneration and weakness. However, patients succumb from dilated cardiomyopathy and respiratory failure (McNally et al., 2015), usually before the age of 30 years. In the heart, DMD manifests as electrocardiogram abnormalities, diastolic dysfunction, fibrosis, systolic dysfunction and fibrosis, which culminate in heart failure (Finsterer and Cripe, 2014). AAV-mediated gene therapy and gene-editing strategies show great promise in animal models (Amoasii et al., 2018; Chamberlain and Chamberlain, 2017). Yet, to date, DMD patients receive non-specific heart failure treatments, beta-blockers, or ACE inhibitors, largely due to a lack of understanding of the mechanisms underlying the cardiac failure.

We previously uncovered an unexpected association of dystrophin deficiency with short telomeres, the hexanucleotide TTAGGG repeats that cap and protect the ends of chromosomes. This stemmed from experiments directed at resolving the conundrum that the mdx^{4cv} mouse model, which lacks dystrophin as in DMD patients but has a normal lifespan, does not manifest the cardiac disease from which patients succumb. Strikingly, human telomeres (~10 kb) are significantly shorter than mouse telomeres (>40 kb). This led us to test our hypothesis that "humanizing" telomere lengths in the mdx^{4cv} mouse might reveal a disease phenotype with greater fidelity to human DMD. Indeed, we found that the dilated cardiomyopathy phenotype was faithfully recapitulated in mdx^{4cv}/mTR^{G2} mice in which telomere lengths were shortened by breeding of the mdx^{4cv} mouse with the mouse TERC knockout (mTR), the RNA component of telomerase (Chang et al., 2016; Mourkioti et al., 2013). Moreover, cardiomyocytes in murine mdx^{4cv}/mTR^{G2} and in DMD patient cardiac tissues exhibited ~50% reduction in telomere

This is an open access article under the CC BY license (http://creativecommons.org/licenses/by/4.0/).

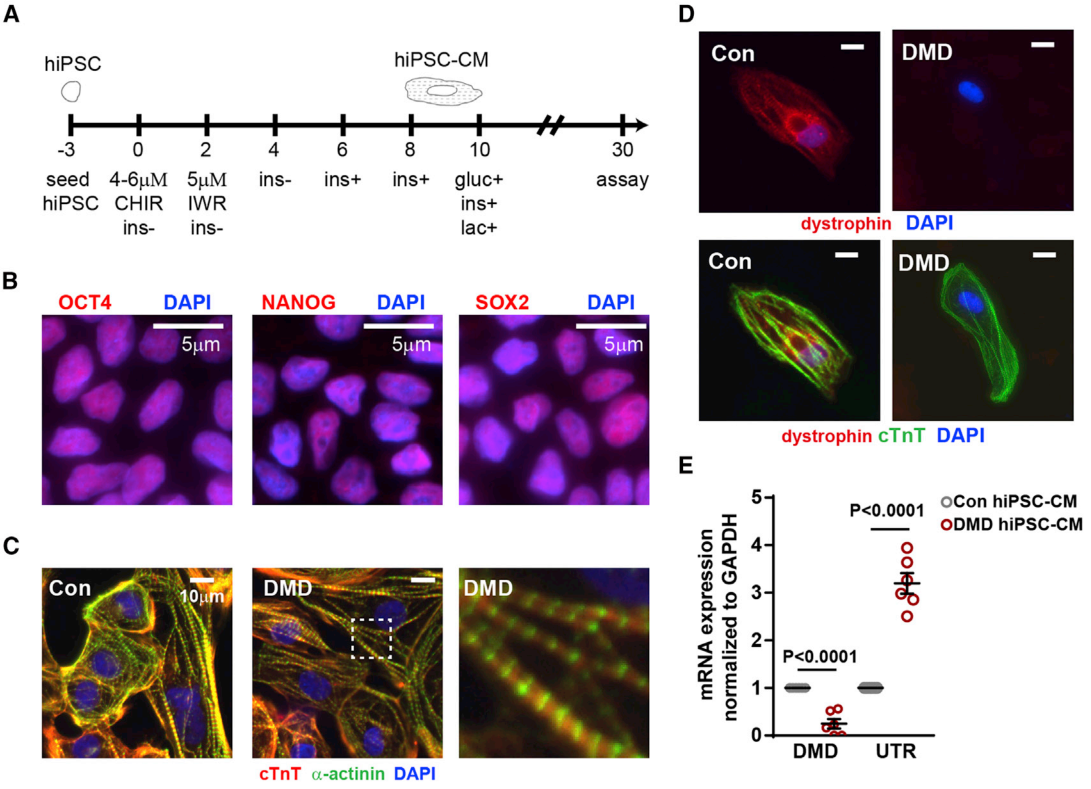

Figure 1. Generation of DMD hiPSC-CMs

(A) hiPSC differentiation protocol for the generation of hiPSC-CMs. CHIR, CHIR-99021; IWR, IWR-1; ins, insulin; gluc, glucose; lac, lactate. hiPSCs were differentiated in medium supplemented with B27 minus insulin (ins). On day 6, the medium was supplemented with B27, including insulin. On day 10, hiPSC-CMs were cultured in medium without glucose supplemented with B27 and lactate.
(B) Representative micrograph in which hiPSCs were stained with pluripotent stem cell markers OCT4, NANOG, and SOX2.
(C) Representative micrograph in which hiPSC-CMs were stained with cardiac troponin T, α-actinin, and DAPI.
(D) Representative micrograph in which healthy and DMD hiPSC-CMs were stained with dystrophin, cardiac troponin T, and DAPI.
(E) Endogenous dystrophin (DMD) and utrophin (UTR) expression levels were determined by qRT-PCR in hiPSC-CMs (n = 6 independent experiments). Data shown as the mean ± SEM. Student's t test was used to calculate significance.

signal as measured by quantitative fluorescence *in situ* hybridization (Q-FISH) relative to mdx^{4cv}/mTR^{Het} mouse controls or cardiomyocytes in age-matched human controls (Chang et al., 2016; Mourkioti et al., 2013). However, the mechanism by which this proliferation-independent telomere shortening is triggered remains unknown.

To gain insight into the molecular and cellular etiology of telomere shortening, here we model human DMD cardiomyopathy in culture and chart disease progression using cardiomyocytes differentiated from patient and isogenic control human induced pluripotent stem cells (hiPSC-CMs), a robust system for studying cardiac defects (Karakikes et al., 2015; Wu et al., 2019b). Importantly, we establish a bioengineered platform that allows for cardiomyocyte orientation with a 1:7 aspect ratio and mimics the stiffness of the DMD fibrotic heart. We show that DMD hiPSC-CMs exhibit aberrant calcium handling, contractile defects, and proliferation-independent telomere shortening. Furthermore, this telomere shortening is post-mitotic, progressive, and contraction dependent. By culturing hiPSC-CMs on a bioengineered platform that mimics the stiffness of healthy and fibrotic myocardium, we provide evidence that contractile dysfunction is exacerbated on a stiff myocardium characteristic of the DMD heart.

RESULTS

Duchenne hiPSC-CMs recapitulate pathogenic features of dilated cardiomyopathy

To study the molecular underpinnings of telomere shortening in DMD hiPSC-CMs, we differentiated six DMD and six control hiPSC lines into beating cardiomyocytes using well-established protocols (Burridge et al., 2015) (Table S1; Figure 1A). Our studies utilized hiPSC lines from four different laboratories derived from disparate cell types,

including two isogenic pairs in which dystrophin mutations were corrected by CRISPR-Cas9 (Con1 and 2 matching DMD1 and 2) (Dick et al., 2013), and one isogenic pair in which a deletion of the first six exons was introduced into healthy cells by CRISPR-Cas9 (Con3 matching DMD3) (Guan et al., 2014; Pioner et al., 2020), three nonfamilial healthy controls (Con4, 5, and 6), and three DMD lines (DMD4, 5, and 6) (Table S1). Pluripotency markers for hiPSC were evaluated by immunofluorescence staining for pluripotency markers OCT4, NANOG, and SOX2 (Figure 1B), and beating cardiomyocytes were determined to express hallmark proteins, cardiac troponin (cTnT), and α-actinin (Figure 1C). The presence or absence of dystrophin was confirmed by immunofluorescence (Figure 1D) as well as by qRT-PCR (Figure 1E).

To evaluate basal calcium levels, ratiometric Fura2 measurements under 1 Hz pacing conditions were used, as described previously (Wu et al., 2019a). We observed increased varied resting calcium ratio (Figure 2A), decreased trend in peak calcium ratio (Figure 2B), decreased transient amplitude (Figure 2C), no difference in time to peak except Con2/DMD2 (Figure 2D), increased transient duration 90 (Figure 2E), and increased decay tau (Figure 2F). Similar trends were observed for spontaneous calcium transients using the Fluo-4 method as described previously (Wu et al., 2015). Compared with controls, DMD hiPSC-CMs exhibited aberrant calcium transients (Figure S1A) with decreased transient amplitude, with the exception of Con2/DMD2 (Figure S1B), delayed time to peak (Figure S1C), prolonged TD50 (Figure S1D) but no difference in decay tau (Figure S1E), similar to previously characterized calcium transients in DMD (Guan et al., 2014; Pioner et al., 2020) and other genetic dilated cardiomyopathy hiPSC-CMs (Sun et al., 2012; Wu et al., 2015). Together, our results confirm that DMD hiPSC-CMs exhibited aberrant calcium handling using two different calcium transient imaging methods, while showing good line to line consistency.

DMD hiPSC-CMs exhibit dysfunctional contractile force generation when subjected to increased mechanical load

To measure DMD hiPSC-CM contractile force, we developed a traction force microscopy platform that mimics the stiffness of the heart tissue before (10 kPa) and after the onset of fibrosis (35 kPa) (Engler et al., 2008) using a tunable hydrogel (Figure 3A) (Ribeiro et al., 2015). Single hiPSC-CMs were configured by micropatterns to assume a physiological 7:1 length:width aspect ratio, characteristic of adult cardiomyocytes and previously shown to promote hiPSC-CM sarcomere alignment and maturation (Ribeiro et al., 2015). Our traction force microscopy platform captures the force generated by single cardiomyocytes as a function of hydrogel deformation measured by tracking the displacement of embedded fluorescent beads (Figure 3B), and traction stress is then reconstructed using Fourier transform traction cytometry. The contractile parameters, including force and velocity, were measured in several contraction cycles in DMD and healthy hiPSC-CMs cultured on 10 and 35 kPa hydrogel devices (Figure 3C, Videos S1–S6).

A significant decrease in force generation was observed in DMD hiPSC-CMs compared with controls on 35 kPa, but not on 10 kPa hydrogels (Figure 3D). DMD hiPSC-CMs on 35 kPa hydrogel devices also exhibited a decrease in contraction velocity (Figure 3E), relaxation velocity (Figure 3F), and cell surface area (Figure 3G), which is consistent with our calcium-handling measurements. The fact that contractile deficiency was only apparent in DMD hiPSC-CMs cultured on 35 kPa substrates suggests that DMD cardiomyocytes are unable to compensate for dystrophin deficiency once the heart tissues undergo fibrotic stiffening, a hallmark of DMD cardiomyopathy.

Under basal conditions, DMD hiPSC-CMs exhibit increased reactive oxygen species (ROS) (Figure S2A), but no difference in mitochondrial respiration was detected using the Seahorse bioanalyzer (Figures S2B and S2C). However, it is known that cardiomyocytes switch to glycolytic metabolism in heart failure conditions (Bertero and Maack, 2018). Under nutrient starvation (Figure S2D), glucose only (Figure S2E), pyruvate only (Figure S2F), or palmitate only (Figure S2G) conditions, DMD hiPSC-CMs exhibit a significant decrease in basal oxygen consumption rate and maximal oxygen consumption rate. Together, these data provide strong evidence that DMD hiPSC-CMs exhibit metabolic maladaptation and contractile dysfunction similar to that seen in DMD heart failure progression.

Telomere shortening in the absence of cell division

We sought to determine if DMD hiPSC-CMs would exhibit telomere shortening similar to that seen in cardiomyocytes of human DMD cardiac tissues (Chang et al., 2018) and mdx^{4cv}/mTR^{G2} cardiac tissues (Chang et al., 2016). To measure telomeres, we used Q-FISH and measured the telomere signal of proliferative hiPSCs and differentiated hiPSC-CMs over a time course of differentiation (Figures 4A and 4B). Telomere signals of proliferating hiPSCs did not differ statistically for healthy controls and DMD hiPSCs, or for the three isogenic pairs (Figures 4C and S3A).

Telomere loss is typically a "passive" process that accompanies incomplete replication at each cell division in the course of aging (Blackburn et al., 2015; López-Otín et al., 2013). The hiPSC-CM platform allowed us to test if cardiomyocytes can undergo telomere shortening in the absence of DNA replication and cell division. This is of particular interest since cardiomyocytes are largely post-mitotic in mice and humans during postnatal development (Bergmann et al., 2015; Porrello et al., 2011). To address this possibility,

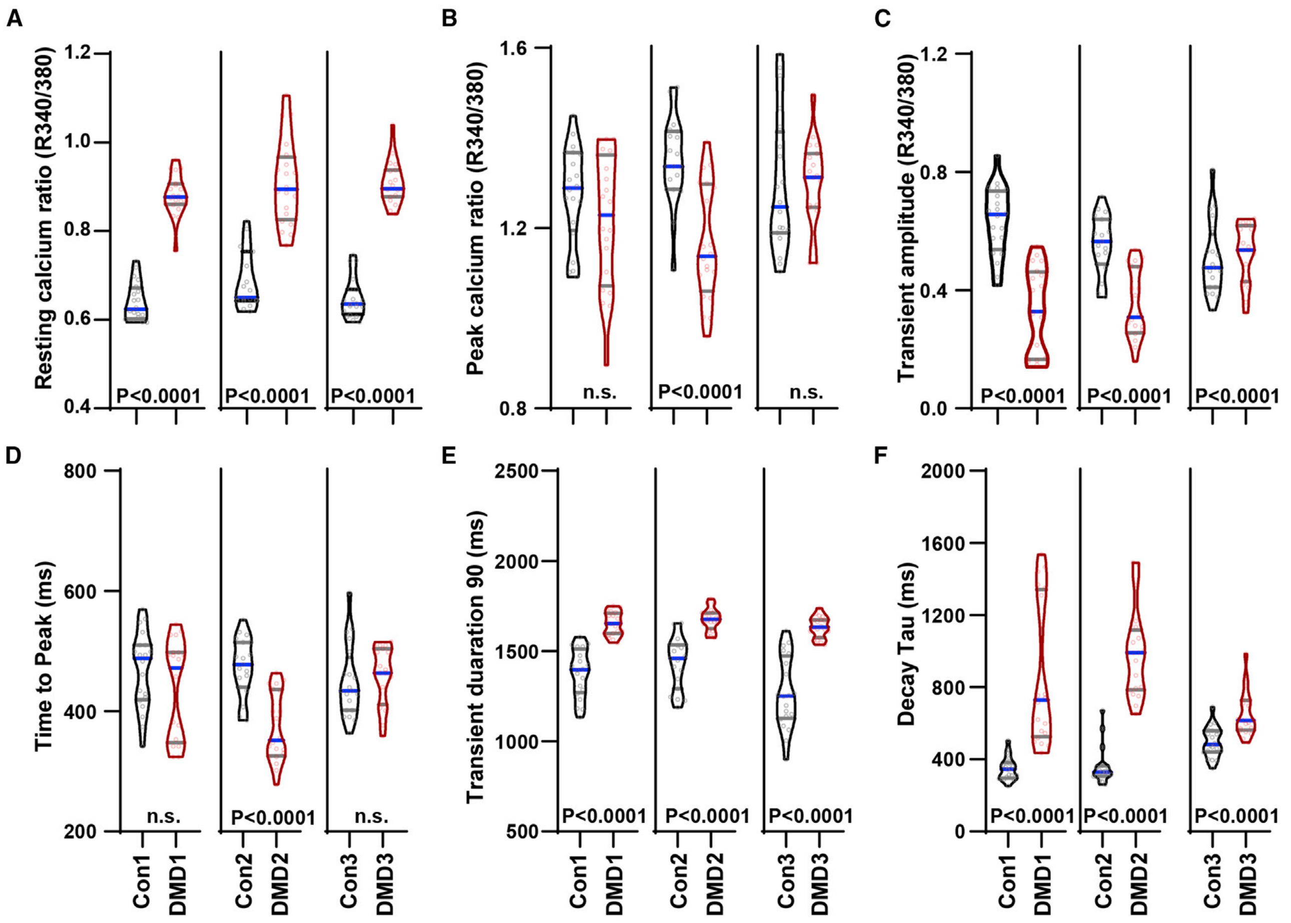

Figure 2. DMD hiPSC-CMs exhibit aberrant calcium-handling properties

Using ratiometric-based Fura2 calcium imaging, (A) resting calcium ratio, (B) peak calcium ratio, (C) transient amplitude, (D) time to peak, (E) transient duration 90, and (F) decay tau per isogenic hiPSC pairs were plotted (isogenic pairs only; n = 3 independent experiments, 19–20 cells analyzed). Data are shown as violin plots where blue median and gray quartiles are shown. Student's t test was used to calculate significance.

we assessed telomere lengths in conjunction with DNA replication measured by EdU (5-ethynyl-2′-deoxyuridine) incorporation (24 h pulse) over a time course of hiPSC-CM differentiation, where day 0 is defined as the first day of differentiation and each day thereafter is the time point post induction of differentiation (Figures 4D, 4E, and S4A). In agreement with our DMD mouse (mdx^{4cv}/mTR^{G2}) and DMD patient results (Chang et al., 2016; Mourkioti et al., 2013), we observed progressive telomere shortening, reaching 50% telomere reduction by day 30 in DMD hiPSC-CMs compared with controls (Figures 4D and S4A). EdU labeling revealed that proliferation is still occurring at day 16 but ceases in hiPSC-CMs between days 20 and 30 (Figures 4E, S4B, and S4C). Interestingly, it was during this post-mitotic period that we observed a progressive telomere loss in DMD hiPSC-CMs compared with controls culminating in a ~50% reduction by day 30 (Figures 4D and S4A).

We postulated that an early molecular trigger of telomere shortening could be telomere deprotection via loss of the shelterin proteins that cap the telomeres. To address this possibility, we isolated cardiomyocytes on day 20, at the onset of proliferation-independent telomere shortening, using flow cytometric enrichment for hiPSC-CMs based on mitochondrial membrane potential assessed by TMRM staining, as described previously (Chang et al., 2018). qRT-PCR revealed decreased transcript levels of telomere repeat binding proteins (*TRF1* and *TRF2*) and shelterin complex proteins (*RAP1*, *TIN2*, and *POT1*) in DMD relative to control hiPSC-CMs (Figure S3D). To determine whether shortened telomeres led to the activation of the DNA damage response, we assessed the levels of p53 (Figure 5A), the number of the p53 binding protein 1 (53BP1) foci per single cell (Figures 5B and 5D), and p21 levels (Figures 5C and 5E). We found that p53 protein was upregulated in DMD hiPSC-CMs

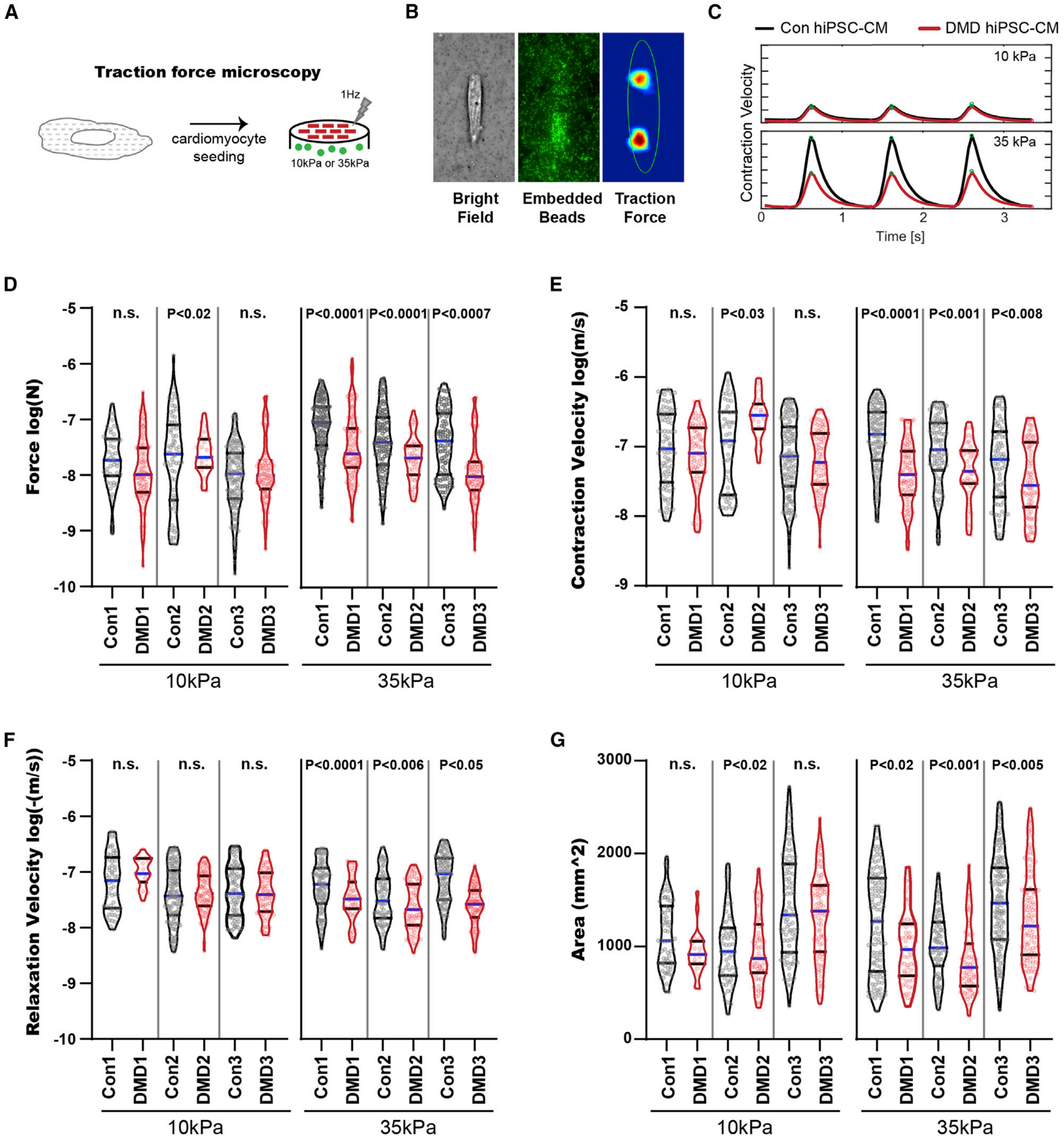

Figure 3. DMD hiPSC-CMs exhibit contractile dysfunction under fibrotic microenvironment challenge

(A) Contractile assessment of DMD hiPSC-CMs using traction force microscopy where hiPSC-CMs were seeded onto micropatterned tunable hydrogel devices.

(B and C) (B) Representative micrographs of contraction force generated by a single hiPSC-CM on 10 or 35 kPa hydrogel (bright field, GFP fluorescent beads, Fourier traction force cytometry) and (C) representative contraction cycles are shown.

(D–G) (D) Force, (E) contraction velocity, (F) relaxation velocity, and (G) cell area of single Con and DMD hiPSC-CMs subjected to 10 and 35 kPa hydrogels were measured (n = 3 independent experiments, 19–143 cells analyzed). Data are shown as violin plots where blue median and gray quartiles are shown. Student's t test was used to calculate significance.

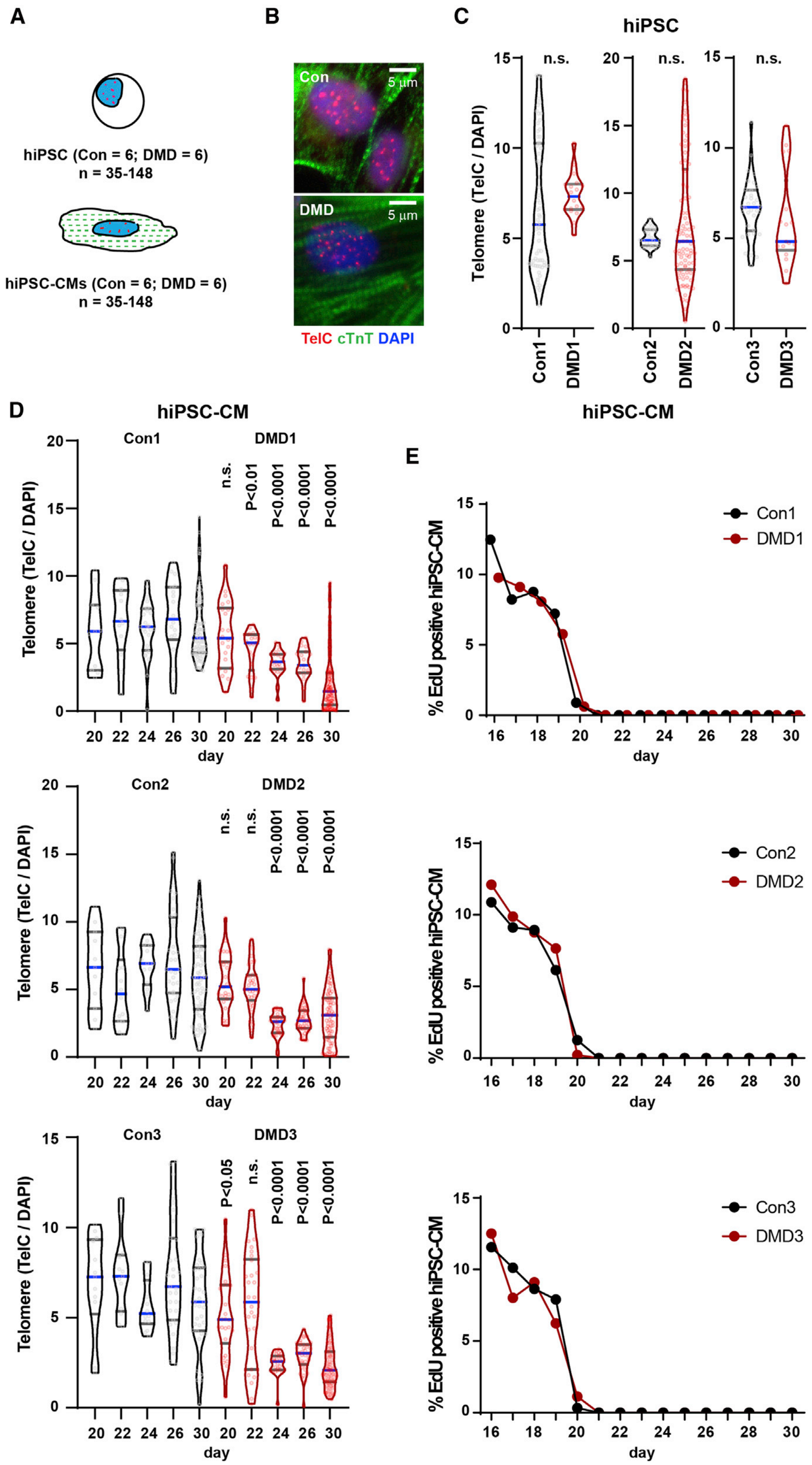

(legend on next page)

(Figure 5A) and that the incidence of 53BP1-positive hiPSC-CMs increased from 10% to 50% in control and DMD, respectively (Figures 5D and S4E). However, we did not detect a significant difference in day 30 DMD hiPSC-CMs compared with controls when we scored cells with telomere-induced foci defined as more than three 53BP1 foci colocalized with telomere probe (Figure S3E) (Takai et al., 2003). Furthermore, this 53BP1 accumulation is correlated with the expression of p53 downstream target p21 protein (Figures 5C and 5E). Activation of the DNA damage response, as indicated by elevated 53BP1, induces p53-mediated repression of PGC-1α, which in turn impedes mitochondrial biogenesis (Figures 5F–5H). Moreover, we observed an increase in apoptotic markers caspase-3 (Figure S3F), cleaved PARP (Figure S3G), and accumulation of β-galactosidase signal in DMD hiPSC-CMs compared with healthy controls (Figure S3H). Interestingly, when we blocked DMD hiPSC-CM contraction using blebbistatin daily starting at day 20, with a dose that locks the myosin heads in a low affinity state, thus preventing actin binding (Skwarek-Maruszewska et al., 2009), telomere shortening was abrogated, as determined by measurements on day 30 (Figure 5I). Here, our data show shelterin gene downregulation, with concomitant telomere shortening independent of cell division due to aberrant contraction, elicits a p53-dependent DNA damage response.

DISCUSSION

We provide evidence for progressive telomere shortening via a previously unrecognized replication-independent mechanisms in hiPSC-derived cardiomyocytes that harbor mutations in the structural protein, dystrophin. This is remarkable, as beyond early postnatal development, the heart is a relatively non-proliferative organ (Bergmann et al., 2015; Porrello et al., 2011). We demonstrated previously that humanizing the telomere lengths unmasks the dilated cardiomyopathy phenotype in the mouse model of DMD (mdx^{4cv}) (Chang et al., 2016; Mourkioti et al., 2013) as well as bicuspid valve phenotype in Notch haploinsufficient ($Notch^{+/-}$) mice (Theodoris et al., 2017). Telomere attrition occurs “passively” in proliferative systems due to the end-replication problem during cell division (Arnoult and Karlseder, 2015; Sfeir and de Lange, 2012). Using the DMD hiPSC-disease model, we were able to chart the dynamics of telomere shortening and show that it occurs independent of proliferation in DMD hiPSC-CMs, corroborating and extending snapshots of the pathogenic process previously seen in murine and human cardiac tissues (Chang et al., 2016, 2018; Mourkioti et al., 2013).

The DMD hiPSC-CM model and the use of traction force microscopy on stiff hydrogels reported here faithfully recapitulates features of cardiomyopathy in DMD patients: aberrant calcium handling and contractile dysfunction (Finsterer and Cripe, 2014), increased ROS production (Chang et al., 2016), and decreased contractile force (Finsterer and Stollberger, 2003; Sasaki et al., 1998). Moreover, our results are in agreement with dilated cardiomyopathy phenotypes seen in other genetic etiologies, e.g., troponin T deficiency (Sun et al., 2012; Wu et al., 2015). Importantly, we provide insights into the mechanism by which an increased stiffness following the development of fibrosis in the DMD myocardium at later stages compromises the contractile function of DMD hiPSC-CMs. Our results suggest that cardiomyocytes can compensate for the lack of dystrophin in the healthy heart (10 kPa), but not the stiff, fibrotic heart (35 kPa), which could explain why DMD patients become increasingly susceptible to arrhythmia and heart failure as they age. Our characterization of the contractile response to microenvironments of different rigidities highlights the development of an acute mechanical dysfunction in DMD cardiomyocytes in conjunction with the progression of fibrosis in the heart.

The robustness of our findings is underscored by the fact that we used hiPSC lines from six DMD and six controls, generated in four different labs from four diverse starting cell types, including two isogenic lines comprised of CRISPR-corrected DMD mutations and one isogenic line that was a CRISPR-created mutant. In agreement with these reports, irrespective of hiPSC origin, DMD or control, we detected similar markedly long telomere lengths at the outset in hiPSCs. Strikingly, upon differentiation into cardiomyocytes, DMD hiPSC-CMs exhibited a dramatic reduction in telomere lengths relative to controls, affording a cell model to study and monitor telomere shortening in DMD cardiomyocytes. Our observations corroborate the marked telomere reduction that we and others previously

Figure 4. DMD hiPSC-CMs exhibit telomere shortening and DNA damage response

(A) Telomere length (TelC) was quantified by immunofluorescence staining (Q-FISH) relative to nuclear DAPI staining for hiPSC and hiPSC-CMs.

(B) Representative micrographs of hiPSC-CMs stained with cardiac troponin T, TelC and DAPI are shown.

(C) Quantification of hiPSC telomere using Q-FISH (n = 3 independent experiments, 17–86 cells analyzed).

(D) Telomere Q-FISH reveals progressive telomere loss in DMD hiPSC-CMs between days 20 and 30 (n = 3 independent experiments, 11–179 cells analyzed).

(E) hiPSC-CMs were devoid of EdU between days 20 and 30. Data are shown as violin plots where blue median and gray quartiles are shown. Student’s t test was used to calculate significance.

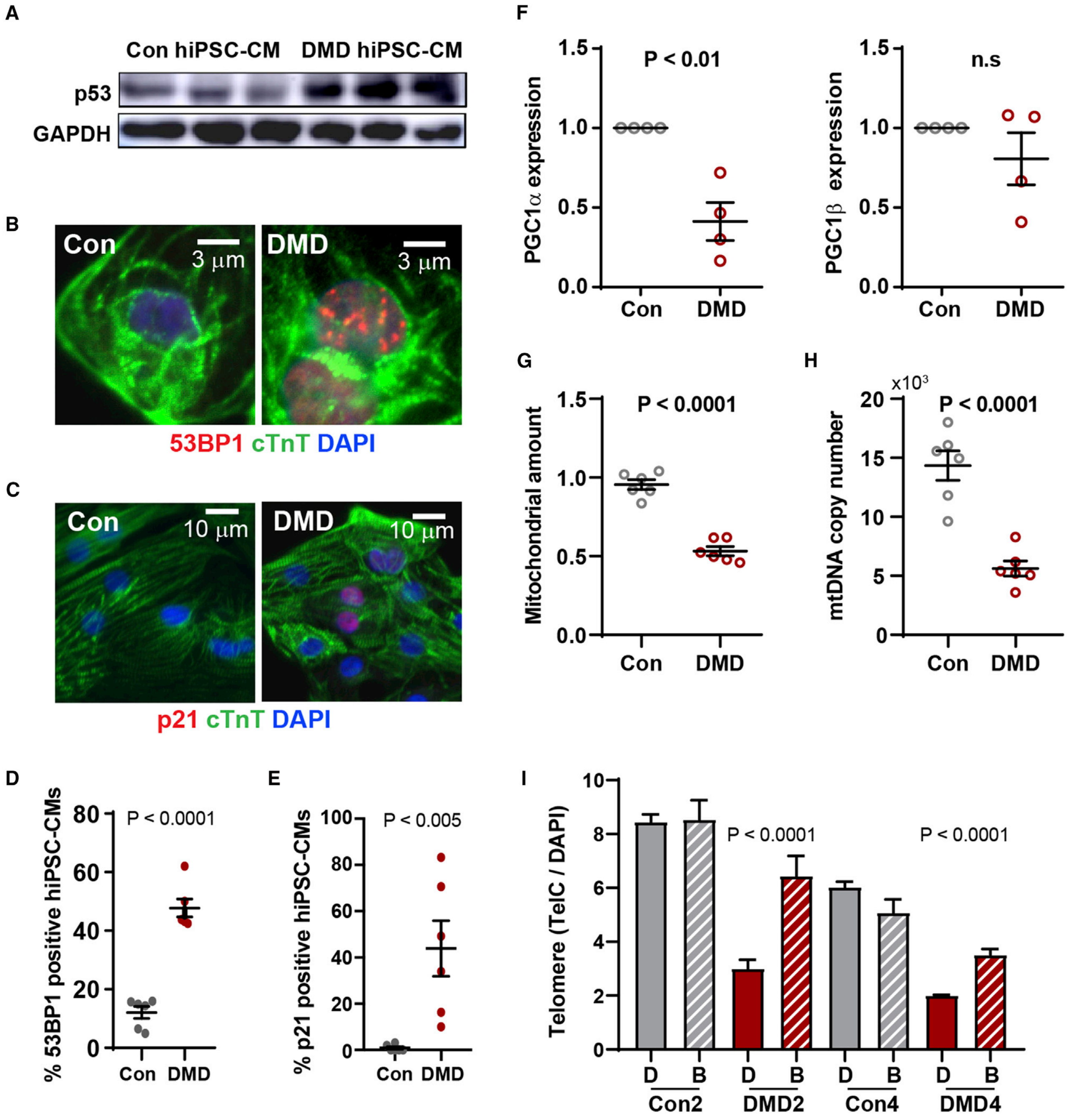

Figure 5. DMD hiPSC-CMs exhibit p53 upregulation

(A–E) Representative micrographs of (A) p53 activation by immunoblotting (n = 3 independent experiments), (B) DNA damage 53BP1 foci by immunofluorescence (n = 6 independent experiments), and (C) p21 (n = 6 independent experiments) in cardiac troponin T+ hiPSC-CMs are shown and (D and E) quantified, respectively.

(F) Reduced expression levels of PGC-1α, master regulator of mitochondrial biogenesis, determined by qRT-PCR (n = 6 independent experiments).

(G and H) (G) Mitochondria amount (n = 6 independent experiments) and (H) mitochondrial copy number (n = 6 independent experiments) in hiPSC-CMs were assessed by MitoTracker Green and qRT-PCR using mitochondrial gene (Nd2) to nuclear DNA (Nrf1) primers, respectively. Data represent mean ± SEM. Student's t was test used for statistical analysis.

(I) Telomere loss prevented when contraction of DMD hiPSC-CMs was inhibited between days 20 and 30 using blebbistatin was quantified (n = 3 independent experiments, 35–1,834 cells analyzed). Data represent mean ± SEM. Student's t test was used for statistical analysis.

detected in cardiomyocytes of genetic cardiomyopathic patients (Chang et al., 2018) or cardiac tissues from patients who succumbed to heart failure (Oh et al., 2003; Terai et al., 2013).

A molecular trigger for telomere shortening appears to be the loss of the shelterin complexes (Sfeir and de Lange, 2012). In accordance, we find that binding of shelterin complexes to the telomeres in DMD cardiomyocytes is reduced at the onset of proliferation-independent telomere shortening, possibly due to the stress of contraction in the absence of dystrophin. Based on the observed ~30% increase in ROS production in DMD hiPSC-CMs and previous findings by others showing high ROS can drive telomere oxidation and disrupt shelterin binding (Opresko et al., 2005), we speculate that direct oxidation of uncapped telomere repeats may also play a role in the observed telomere shortening (von Zglinicki et al., 1995). In agreement, we showed that shelterin expression is reduced in DMD hiPSC-CMs, and others have shown that this occurs in human cardiomyopathy tissue (Oh et al., 2003). However, whether loss of shelterin expression is the cause or consequence of telomere deprotection in DMD cardiomyocytes remains to be determined. Furthermore, ROS production is driven, in part, by contractile activity in DMD cardiomyocytes (Prosser et al., 2011). Our finding that blebbistatin treatment impedes telomere shortening suggests that contractile function plays a crucial role. Whether this is due to ROS-mediated telomere deprotection or disruption of sarcomere organization remains to be elucidated. Nonetheless, these data provide support for our hypothesis that contraction in conjunction with the genetic absence of a key structural contractile protein plays a causal role in telomere shortening, as reported here in the iPSC-CM disease model and previously in the mouse model of DMD (Chang and Blau, 2018). In parallel, loss of structural integrity may induce transcriptional changes, as demonstrated in laminopathic cardiomyocytes (Cheedipudi et al., 2019). Downstream sequelae include induction of a DNA damage response evident by accumulation of 53BP1, p53, and p21 proteins, mitochondrial demise, and metabolic failure in DMD hiPSC-CMs, in agreement with mTR^{G4} mouse models of aging (Sahin et al., 2011) and the DMD mouse model with “humanized” telomeres (mdx^{4cv}/mTR^{G2}) (Chang et al., 2016).

The acquisition of a “senescence-like state” by DMD hiPSC-CMs is suggested by the expression of 53BP1, p53, p21, and β-galactosidase, as recently described in other non-dividing systems (Gorgoulis et al., 2019). Although the animal models support the importance of telomere length in cardiomyopathy pathogenesis (Chang et al., 2016; Sahin et al., 2011), a prolonged DNA damage response may disrupt other pathways important for cell survival, such as the PGC-1α repression of mitochondrial biogenesis. Whether mitochondrial dysfunction plays a role in the pathogenesis remains to be elucidated (Correia-Melo et al., 2016). We hypothesize that telomere shortening is at play in other stressed post-mitotic differentiated cell types, such as neurons harboring mutations (Ain et al., 2018) or acquired defects (Aguado et al., 2019). Together, our findings provide evidence showing that telomere shortening occurs independent of proliferation and is potentially driven by contraction-induced ROS in dystrophic cardiomyocytes. Our results suggest a novel therapeutic target, telomere maintenance, to ameliorate or halt the progression of DMD cardiomyopathy regardless of mutational status.

EXPERIMENTAL PROCEDURES

Statistics

Statistical differences between patient hiPSC and hiPSC-CM groups were analyzed using one-way analysis of variance by comparing the mean of each group with the mean of every other group, followed by Holm-Sidak’s multiple comparison test (non-isogenic groups) or by two-tailed unpaired Student’s t tests (isogenic pairs). Image capture and quantification analyses were performed in a double-blinded fashion to avoid bias. All data are shown as the mean ± SEM. Significant differences were determined as $p < 0.05$. For a detailed summary of all statistical calculations please see Data S1.

hiPSC culture and differentiation into hiPSC-CMs

All protocols using hiPSC were reviewed and approved by the Stanford Stem Cell Research Oversight committee (#602) as well as the Ethics Review committee at Ninth People’s Hospital, Shanghai Jiao Tong University School of Medicine (2018-207-K32). hiPSCs were grown on Matrigel-coated (Corning, 356231) plates using Nutristem medium. The medium was changed daily, and cells were passaged every 4 days using Accutase (Sigma, A6964) and seeded in 1:8 dilution with addition of 5 μM Y-27632 2HCl (Selleck Chem, S1049). hiPSCs were grown to 70%–90% confluence and subsequently differentiated into beating cardiomyocytes, as demonstrated previously (Chang et al., 2018). Beating hiPSC-CMs were purified against non-cardiomyocytes and matured by culturing in glucose-free conditions using RPMI-1640 medium without glucose with B27 supplement and 5 mM lactate (Life Technologies) until day 30 (Hinson et al., 2015; Ni et al., 2021; Tohyama et al., 2013).

Blebbistatin treatment

Beating day 20 hiPSC-CMs were treated with 5 μm blebbistatin (Abcam, ab120425) to lock the myosin heads in a low affinity state for actin to prevent contraction (Skwarek-Maruszewska et al., 2009) as per the manufacturer’s recommendation. Blebbistatin and DMSO were first mixed with RPMI-1640 medium without glucose with B27 supplement and 5 mM lactate (Life Technologies), the medium was changed daily until day 30. hiPSC-CMs were then fixed for downstream telomere quantification.

Fluorescence-activated cell sorting purification of hiPSC-CMs

Fluorescence-activated cell sorting (FACS) purification was carried out for qRT-PCR data. Using a described previously protocol (Chang et al., 2018; Tohyama et al., 2013), hiPSC-CMs were purified using FACS. Cell cultures were dissociated using Accutase and stained with 50 nM tetramethylrhodamine methyl ester perchlorate (TMRM). Cells were gated for side scatter and forward scatter to avoid debris and doublets and TMRM+ (cardiomyocytes) were isolated for downstream characterization.

Immunofluorescence and Q-FISH

hiPSC-CMs and hiPSCs were fixed with 4% paraformaldehyde in PBS for 5 min at room temperature and subsequently maintained in PBS at 4°C. For the Q-FISH time course, day 18 hiPSC-CMs were seeded onto Matrigel-coated 8-chamber slides and fixed at various days as indicated. Telomere Q-FISH was performed as described previously using TelC-Cy3 PNA probe (CCCTAACCCTAACCCTAA) (PNA Bio, F1002) (Chang et al., 2016; Mourkioti et al., 2013). Samples were blocked with staining buffer (20% fetal bovine serum/0.1% Triton X-100/PBS) and stained with rabbit anti-Oct4 (1:400, Abcam, ab19857), rabbit anti-Sox2 (1:400, Abcam, ab92494), rabbit anti-Nanog (1:400, Abcam, ab80892), rabbit anti-53BP1 (1:400, Bethyl, IHC-00001), p21 Waf1/Cip1 (Cell Signaling Technology, 2947S), rabbit anti-cleaved PARP1 (1:400, Abcam, ab32064), rabbit anti-cleaved caspase-3 Asp175 (1:400, CST, no. 9661), rabbit α-actinin (1:400, Thermo Scientific, 42-1400), rabbit anti-dystrophin (1:100, Abcam, ab15277), and/or mouse anti-cardiac troponin T antibody (1:500, Abcam, ab74275 or ab8295) for 2 h at room temperature or overnight at 4°C in staining buffer, washed, and stained with goat anti-mouse or anti-rabbit Alexa 488, 594, or 647 (1:400, Abcam) for 1 h, washed and counterstained with 1 μg mL^{-1} DAPI in PBS for 5 min, washed with dH_2O, air dried, and mounted with ProLong Gold Antifade (Life Technologies). To assay cell proliferation, hiPSC-CMs were pulsed with 24 h EdU and proliferation was assessed using the Click-iT EdU Alexa Fluor 488 Imaging Kit (Thermo Fisher Scientific). Images were captured on a Nikon Spinning Disk Confocal microscope using a PLAN APO 40x objective. Telomere signal intensity for cardiomyocytes (troponin T+) was determined as PNA signal (TelC probe) in each nucleus normalized to the DAPI intensity of that nucleus using ImageJ plugin Telometer, as described previously (Chang et al., 2016; Meeker et al, 2002, 2004; Mourkioti et al., 2013).

Immunoblotting

Immunoblotting was performed as described previously (Chang et al., 2016). In brief, 30 μg of total RIPA protein lysate was separated on a NuPAGE Bis-Tris 4%–12% gel under MOPS condition (Thermo Scientific), transferred to a PVDF membrane (Sigma-Aldrich), and immunoblotted using mouse anti-p53 (1:2000, ProteinTech, 10442-1-AP) and rabbit anti-GAPDH (1:3,000, CST, no. 5174) antibodies followed by goat anti-mouse (1:10,000, A16072, Sigma-Aldrich) and goat anti-rabbit HRP (1:10,000, G-21234, Sigma-Aldrich).

qRT-PCR

RNAs were extracted using the Direct-zol RNA MiniPrep Plus with Zymo-Spin IIICG Columns (Zymo Research, R2070), the quantity and quality of RNA was determined using a NanoDrop 2000 spectrophotometer (Thermo Fisher Scientific), and cDNA was reverse transcribed using a High-Capacity cDNA Reverse Transcription Kit (Life Sciences/ABI, 4374966). Total DNA were extracted using a DNeasy Blood & Tissue Kit (QIAGEN, 69506) and the quantity and quality of DNA were determined using a NanoDrop 2000 spectrophotometer. mRNA expression levels were detected using TaqMan probes for the following genes: DMD (Hs00758098), UTR (Hs01126016), TRF1 (Hs00819517_mH), TRF2 (Hs01030567_m1), TPP1 (Hs00166099_m1), TIN2 (Hs01554309_g1), RAP1 (Hs00430292_m1), POT1 (Hs00209984_m1), PGC1α (Hs01016719_m1), PGC1β (Hs00991677_m1), and GAPDH (Hs02758991_g1). Fold enrichment was calculated using $2\hat{}$-ΔΔCT normalized to GAPDH, then to healthy controls. Mitochondrial copy number assay was performed as described previously (Evdokimovsky et al., 2011) using the following TaqMan probes: NRF1 (Hs04926189_cn), ND2 (Hs02596874_g1).

Calcium transient, Seahorse bioanalyzer and ROS measurements, and traction force microscopy.

SUPPLEMENTAL INFORMATION

Supplemental information can be found online at https://doi.org/10.1016/j.stemcr.2021.04.018.

AUTHOR CONTRIBUTIONS

A.C.Y.C. and H.M.B. conceived and designed the research. G.P., A.J.S.R., and B.L.P. conceived and designed traction force microscopy. A.C.Y.C., G.P., A.C.H.C., K.K., H. Wu, S.-G.O., A.E., S.A., C.H., J.R., A.J.S.R., E.L., and H. Wang performed the research. A.C.Y.C., G.P., A.C.H.C., K.K., H. Wu., S.A., and H.M.B. analyzed the data. All authors contributed to the writing of the manuscript.

DECLARATION OF INTERESTS

The authors declare no competing interests.

ACKNOWLEDGMENTS

We thank Jon Mulholland and Cedric Espenel for expert microscopy assistance in the Nikon Spinning Disk Confocal microscope (funded jointly by the School of Engineering and the Beckman Center). This research was supported by Shanghai Pujiang Program (19PJ1407000 to A.C.Y.C.); The Program for Professor of Special Appointment (Eastern Scholar) at Shanghai Institutions of Higher Learning (0900000024 to A.C.Y.C.); Innovative Research Team of High-Level Local Universities in Shanghai (to A.C.Y.C.); the American Heart Association (13POST14480004 and 18CDA34110411 to A.C.Y.C., 18POST33960526 to A.E., 18POST34080160 to G.P., 17MERIT33610009 to J.C.W., and 17CSA33590101 to H.M.B.); the Canadian Institutes of Health Research Fellowship (201411MFE-338745-169197 to A.C.Y.C.); the Swiss National Science Foundation (SNSF) Early Postdoc Mobility Fellowship (#P2SKP2_164954 to G.P.); the China Postdoctoral Science Foundation (2019M661557 to H.W.); the British Heart Foundation (SP/15/9/31605, RG/15/6/31436, PG/14/59/31000, RG/14/1/30588, P47352/Center for Regenerative Medicine to C.D.); BIRAX (04BX14CDLG to C.D.); the Medical

Research Council (MR/M017354/1, G0801098 to C.D.); the National Centre for the Replacement, Refinement and Reduction of Animals in Research (NC/K000225/1) through the NC3Rs CRACK-IT (35911-259146 to C.D.); and the Heart Research UK (TRP01/12 to C.D.); the National Institutes of Health (R35 CA197713 to A.J.G. and R01 AG044815 and R01 AR063963 to H.M.B.); the Baxter Foundation and the Li Ka Shing Foundation (to H.M.B).

Received: January 4, 2021
Revised: April 26, 2021
Accepted: April 26, 2021
Published: May 20, 2021

REFERENCES

Aguado, J., Sola-Carvajal, A., Cancila, V., Revêchon, G., Ong, P.F., Jones-Weinert, C.W., Wallén Arzt, E., Lattanzi, G., Dreesen, O., Tripodo, C., et al. (2019). Inhibition of DNA damage response at telomeres improves the detrimental phenotypes of Hutchinson-Gilford progeria syndrome. Nat. Commun. *10*, 4990–5011. https://doi.org/10.1038/s41467-019-13018-3.

Ain, Q., Schmeer, C., Penndorf, D., Fischer, M., Bondeva, T., Förster, M., Haenold, R., Witte, O.W., and Kretz, A. (2018). Cell cycle-dependent and -independent telomere shortening accompanies murine brain aging. Aging (Albany NY) *10*, 3397–3420. https://doi.org/10.18632/aging.101655.

Amoasii, L., Hildyard, J.C.W., Li, H., Sanchez-Ortiz, E., Mireault, A., Caballero, D., Harron, R., Stathopoulou, T.-R., Massey, C., Shelton, J.M., et al. (2018). Gene editing restores dystrophin expression in a canine model of Duchenne muscular dystrophy. Science *8*, eaau1549. https://doi.org/10.1126/science.aau1549.

Arnoult, N., and Karlseder, J. (2015). Complex interactions between the DNA-damage response and mammalian telomeres. Nat. Struct. Mol. Biol. *22*, 859–866. https://doi.org/10.1038/nsmb.3092.

Bergmann, O., Zdunek, S., Felker, A., Salehpour, M., Alkass, K., Bernard, S., Sjostrom, S.L., Szewczykowska, M., Jackowska, T., Dos Remedios, C., Malm, T., et al. (2015). Dynamics of cell generation and turnover in the human heart. Cell *161*, 1566–1575. https://doi.org/10.1016/j.cell.2015.05.026.

Bertero, E., and Maack, C. (2018). Metabolic remodelling in heart failure. Nat. Rev. Cardiol. *15*, 457–470. https://doi.org/10.1038/s41569-018-0044-6.

Blackburn, E.H., Epel, E.S., and Lin, J. (2015). Human telomere biology: a contributory and interactive factor in aging, disease risks, and protection. Science *350*, 1193–1198. https://doi.org/10.1126/science.aab3389.

Burridge, P.W., Holmström, A., and Wu, J.C. (2015). Chemically defined culture and cardiomyocyte differentiation of human pluripotent stem cells. Curr. Protoc. Hum. Genet. *87*, 21.23.1–15. https://doi.org/10.1002/0471142905.hg2103s87.

Chamberlain, J.R., and Chamberlain, J.S. (2017). Progress toward gene therapy for Duchenne muscular dystrophy. Mol. Ther. *25*, 1125–1131. https://doi.org/10.1016/j.ymthe.2017.02.019.

Chang, A.C.Y., and Blau, H.M. (2018). Short telomeres—a hallmark of heritable cardiomyopathies. Differentiation *100*, 31–36. https://doi.org/10.1016/j.diff.2018.02.001.

Chang, A.C.Y., Chang, A.C.H., Kirillova, A., Sasagawa, K., Su, W., Weber, G., Lin, J., Termglinchan, V., Karakikes, I., Seeger, T., et al. (2018). Telomere shortening is a hallmark of genetic cardiomyopathies. Proc. Natl. Acad. Sci. U S A *2012*, 201714538. https://doi.org/10.1073/pnas.1714538115.

Chang, A.C.Y., Ong, S.-G., LaGory, E.L., Kraft, P.E., Giaccia, A.J., Wu, J.C., and Blau, H.M. (2016). Telomere shortening and metabolic compromise underlie dystrophic cardiomyopathy. Proc. Natl. Acad. Sci. U S A https://doi.org/10.1073/pnas.1615340113.

Cheedipudi, S.M., Matkovich, S.J., Coarfa, C., Hu, X., Robertson, M.J., Sweet, M., Taylor, M., Mestroni, L., Cleveland, J., Willerson, J.T., et al. (2019). Genomic reorganization of lamin-associated domains in cardiac myocytes is associated with differential gene expression and DNA methylation in human dilated cardiomyopathy. Circ. Res. *124*, 1198–1213. https://doi.org/10.1161/CIRCRESAHA.118.314177.

Correia-Melo, C., Marques, F.D.M., Anderson, R., Hewitt, G., Hewitt, R., Cole, J., Carroll, B.M., Miwa, S., Birch, J., Merz, A., Rushton, M.D., et al. (2016). Mitochondria are required for pro-ageing features of the senescent phenotype. Embo J. *35*, 724–742. https://doi.org/10.15252/embj.201592862.

Dick, E., Kalra, S., Anderson, D., George, V., Ritson, M., Laval, S., Barresi, R., Aartsma-Rus, A., Lochmüller, H., and Denning, C. (2013). Exon skipping and gene transfer restore dystrophin expression in hiPSC-cardiomyocytes harbouring DMD mutations. Stem Cells Dev. 150127064140000. https://doi.org/10.1089/2013.0135.

Engler, A.J., Carag-Krieger, C., Johnson, C.P., Raab, M., Tang, H.-Y., Speicher, D.W., Sanger, J.W., Sanger, J.M., and Discher, D.E. (2008). Embryonic cardiomyocytes beat best on a matrix with heart-like elasticity: scar-like rigidity inhibits beating. J. Cell Sci. *121*, 3794–3802. https://doi.org/10.1242/jcs.029678.

Evdokimovsky, E.V., Ushakova, T.E., Kudriavtcev, A.A., and Gaziev, A.I. (2011). Alteration of mtDNA copy number, mitochondrial gene expression and extracellular DNA content in mice after irradiation at lethal dose. Radiat. Environ. Biophys. *50*, 181–188. https://doi.org/10.1007/s00411-010-0329-6.

Finsterer, J., and Cripe, L. (2014). Treatment of dystrophin cardiomyopathies. Nat. Rev. Cardiol. *11*, 168–179. https://doi.org/10.1038/nrcardio.2013.213.

Finsterer, J., and Stollberger, C. (2003). The heart in human dystrophinopathies. Cardiology *99*, 1–19. https://doi.org/10.1159/000068446.

Gorgoulis, V., Adams, P.D., Alimonti, A., Bennett, D.C., Bischof, O., Bishop, C., Campisi, J., Collado, M., Evangelou, K., Ferbeyre, G., Gil, J., et al. (2019). Cellular senescence: defining a path forward. Cell *179*, 813–827. https://doi.org/10.1016/j.cell.2019.10.005.

Guan, X., Mack, D.L., Moreno, C.M., Strande, J.L., Mathieu, J., Shi, Y., Markert, C.D., Wang, Z., Liu, G., Lawlor, M.W., et al. (2014). Dystrophin-deficient cardiomyocytes derived from human urine: new biologic reagents for drug discovery. Stem Cell Res. *12*, 467–480. https://doi.org/10.1016/j.scr.2013.12.004.

Hinson, J.T., Chopra, A., Nafissi, N., Polacheck, W.J., Benson, C.C., Swist, S., Gorham, J., Yang, L., Schafer, S., Sheng, C.C., et al. (2015). HEART DISEASE. Titin mutations in iPS cells define sarcomere insufficiency as a cause of dilated cardiomyopathy. Science *349*, 982–986. https://doi.org/10.1126/science.aaa5458.

Karakikes, I., Ameen, M., Termglinchan, V., and Wu, J.C. (2015). Human induced pluripotent stem cell-derived cardiomyocytes: insights into molecular, cellular, and functional phenotypes. Circ. Res. *117*, 80–88. https://doi.org/10.1161/CIRCRESAHA.117.305365.

López-Otín, C., Blasco, M.A., Partridge, L., Serrano, M., and Kroemer, G. (2013). The hallmarks of aging. Cell *153*, 1194–1217. https://doi.org/10.1016/j.cell.2013.05.039.

McNally, E.M., Kaltman, J.R., Benson, D.W., Canter, C.E., Cripe, L.H., Duan, D., Finder, J.D., Groh, W.J., Hoffman, E.P., Judge, D.P., et al. (2015). Contemporary cardiac issues in Duchenne muscular dystrophy. Working Group of the National Heart, Lung, and Blood Institute in collaboration with Parent Project Muscular Dystrophy. Circulation *131*, 1590–1598. https://doi.org/10.1161/CIRCULATIONAHA.114.015151.

Meeker, A.K., Gage, W.R., Hicks, J.L., Simon, I., Coffman, J.R., Platz, E.A., March, G.E., and De Marzo, A.M. (2002). Telomere length assessment in human archival tissues: combined telomere fluorescence in situ hybridization and immunostaining. Am. J. Pathol. *160*, 1259–1268. https://doi.org/10.1016/S0002-9440(10)62553-9.

Meeker, A.K., Hicks, J.L., Gabrielson, E., Strauss, W.M., De Marzo, A.M., and Argani, P. (2004). Telomere shortening occurs in subsets of normal breast epithelium as well as in situ and invasive carcinoma. Am. J. Pathol. *164*, 925–935. https://doi.org/10.1016/S0002-9440(10)63180-X.

Mourkioti, F., Kustan, J., Kraft, P., Day, J.W., Zhao, M.-M., Kost-Alimova, M., Protopopov, A., DePinho, R.A., Bernstein, D., Meeker, A.K., and Blau, H.M. (2013). Role of telomere dysfunction in cardiac failure in Duchenne muscular dystrophy. Nat. Cell Biol. *15*, 895–904. https://doi.org/10.1038/ncb2790.

Ni, X., Xu, K., Zhao, Y., Li, J., Wang, L., Yu, F., and Li, G. (2021). Single-cell analysis reveals the purification and maturation effects of glucose starvation in hiPSC-CMs. Biochem Biophys Res. Commun. *534*, 367–373. https://doi.org/10.1016/j.bbrc.2020.11.076.

Oh, H., Wang, S.C., Prahash, A., Sano, M., Moravec, C.S., Taffet, G.E., Michael, L.H., Youker, K.A., Entman, M.L., and Schneider, M.D. (2003). Telomere attrition and Chk2 activation in human heart failure. Proc. Natl. Acad. Sci. U S A *100*, 5378–5383. https://doi.org/10.1073/pnas.0836098100.

Opresko, P.L., Fan, J., Danzy, S., Wilson, D.M., and Bohr, V.A. (2005). Oxidative damage in telomeric DNA disrupts recognition by TRF1 and TRF2. Nucleic Acids Res. *33*, 1230–1239. https://doi.org/10.1093/nar/gki273.

Pioner, J.M., Guan, X., Klaiman, J.M., Racca, A.W., Pabon, L., Muskheli, V., Macadangdang, J., Ferrantini, C., Hoopmann, M.R., Moritz, R.L., et al. (2020). Absence of full-length dystrophin impairs normal maturation and contraction of cardiomyocytes derived from human-induced pluripotent stem cells. Cardiovasc. Res. *116*, 368–382. https://doi.org/10.1093/cvr/cvz109.

Porrello, E.R., Mahmoud, A.I., Simpson, E., Hill, J.A., Richardson, J.A., Olson, E.N., and Sadek, H.A. (2011). Transient regenerative potential of the neonatal mouse heart. Science *331*, 1078–1080. https://doi.org/10.1126/science.1200708.

Prosser, B.L., Ward, C.W., and Lederer, W.J. (2011). X-ROS signaling: rapid mechano-chemo transduction in heart. Science *333*, 1440–1445. https://doi.org/10.1126/science.1202768.

Ribeiro, A.J.S., Ang, Y.-S., Fu, J.-D., Rivas, R.N., Mohamed, T.M.A., Higgs, G.C., Srivastava, D., and Pruitt, B.L. (2015). Contractility of single cardiomyocytes differentiated from pluripotent stem cells depends on physiological shape and substrate stiffness. Proc. Natl. Acad. Sci. U S A *112*, 12705–12710. https://doi.org/10.1073/pnas.1508073112.

Sahin, E., Colla, S., Liesa, M., Moslehi, J., Muller, F.L., Guo, M., Cooper, M., Kotton, D., Fabian, A.J., Walkey, C., et al. (2011). Telomere dysfunction induces metabolic and mitochondrial compromise. Nature *470*, 359–365. https://doi.org/10.1038/nature09787.

Sasaki, K., Sakata, K., Kachi, E., Hirata, S., Ishihara, T., and Ishikawa, K. (1998). Sequential changes in cardiac structure and function in patients with Duchenne type muscular dystrophy: a two-dimensional echocardiographic study. Am. Heart J. *135*, 937–944.

Sfeir, A., and de Lange, T. (2012). Removal of shelterin reveals the telomere end-protection problem. Science *336*, 593–597. https://doi.org/10.1126/science.1218498.

Skwarek-Maruszewska, A., Hotulainen, P., Mattila, P.K., and Lappalainen, P. (2009). Contractility-dependent actin dynamics in cardiomyocyte sarcomeres. J. Cell Sci. *122*, 2119–2126. https://doi.org/10.1242/jcs.046805.

Sun, N., Yazawa, M., Liu, J., Han, L., Sanchez-Freire, V., Abilez, O.J., Navarrete, E.G., Hu, S., Wang, L., Lee, A., et al. (2012). Patient-specific induced pluripotent stem cells as a model for familial dilated cardiomyopathy. Sci. Transl Med. *4*, 130ra47. https://doi.org/10.1126/scitranslmed.3003552.

Takai, H., Smogorzewska, A., and de Lange, T. (2003). DNA damage foci at dysfunctional telomeres. Curr. Biol. *13*, 1549–1556. https://doi.org/10.1016/s0960-9822(03)00542-6.

Terai, M., Izumiyama-Shimomura, N., Aida, J., Ishikawa, N., Sawabe, M., Arai, T., Fujiwara, M., Ishii, A., Nakamura, K.-I., and Takubo, K. (2013). Association of telomere shortening in myocardium with heart weight gain and cause of death. Sci. Rep. *3*, 2401. https://doi.org/10.1038/srep02401.

Theodoris, C.V., Mourkioti, F., Huang, Y., Ranade, S.S., Liu, L., Blau, H.M., and Srivastava, D. (2017). Long telomeres protect against age-dependent cardiac disease caused by NOTCH1 haploinsufficiency. J. Clin. Invest. *127*. https://doi.org/10.1172/JCI90338.

Tohyama, S., Hattori, F., Sano, M., Hishiki, T., Nagahata, Y., Matsuura, T., Hashimoto, H., Suzuki, T., Yamashita, H., Satoh, Y., et al. (2013). Distinct metabolic flow enables large-scale purification of mouse and human pluripotent stem cell-derived cardiomyocytes. Cell Stem Cell *12*, 127–137. https://doi.org/10.1016/j.stem.2012.09.013.

Wu, H., Lee, J., Vincent, L.G., Wang, Q., Gu, M., Lan, F., Churko, J.M., Sallam, K.I., Matsa, E., Sharma, A., et al. (2015). Epigenetic regulation of phosphodiesterases 2A and 3A underlies compromised β-adrenergic signaling in an iPSC model of dilated

cardiomyopathy. Cell Stem Cell *17*, 89–100. https://doi.org/10.1016/j.stem.2015.04.020.

Wu, H., Yang, H., Rhee, J.-W., Zhang, J.Z., Lam, C.K., Sallam, K., Chang, A.C.Y., Ma, N., Lee, J., Zhang, H., et al. (2019a). Modelling diastolic dysfunction in induced pluripotent stem cell-derived cardiomyocytes from hypertrophic cardiomyopathy patients. Eur. Heart J. *40*, 3685–3695. https://doi.org/10.1093/eurheartj/ehz326.

Wu, J.C., Garg, P., Yoshida, Y., Yamanaka, S., Gepstein, L., Hulot, J.-S., Knollmann, B.C., and Schwartz, P.J. (2019b). Towards precision medicine with human iPSCs for cardiac channelopathies. Circ. Res. *125*, 653–658. https://doi.org/10.1161/CIRCRESAHA.119.315209.

von Zglinicki, T., Saretzki, G., Döcke, W., and Lotze, C. (1995). Mild hyperoxia shortens telomeres and inhibits proliferation of fibroblasts: a model for senescence? Exp. Cell Res. *220*, 186–193. https://doi.org/10.1006/excr.1995.1305.

Stem Cell Reports, Volume *16*

Supplemental Information

Increased tissue stiffness triggers contractile dysfunction and telomere shortening in dystrophic cardiomyocytes

Alex C.Y. Chang, Gaspard Pardon, Andrew C.H. Chang, Haodi Wu, Sang-Ging Ong, Asuka Eguchi, Sara Ancel, Colin Holbrook, John Ramunas, Alexandre J.S. Ribeiro, Edward L. LaGory, Honghui Wang, Kassie Koleckar, Amato Giaccia, David L. Mack, Martin K. Childers, Chris Denning, John W. Day, Joseph C. Wu, Beth L. Pruitt, and Helen M. Blau

Online Data Supplements

Supplemental Experimental Procedures

Calcium transient

For calcium transient measurement, hiPSC-CMs were disassociated by Accutase and seeded in Matrigel-coated 8-well LAB-TEK® II cover glass imaging chambers (Thermo Fisher) at a density of 20,000 cells per well. Ratiometric calcium, Fura2, imaging was performed as previously described (H. Wu et al., 2019). After recovery, cells were loaded with 5 μM Fluo-4 AM in Tyrode's solution (140 mM NaCl, 5.4 mM KCl, 1 mM MgCl2, 10 mM glucose, 1.8 mM CaCl2, and 10 mM HEPES, pH 7.4 adjusted with NaOH at RT) for 10 min in 37°C incubator. Cells were then washed with pre-warmed Tyrode's solution for 3 times. Spontaneous calcium transient was sampled by confocal microscope (Carl Zeiss, LSM 510 Meta, Göttingen, Germany) with a 63X oil immersed objective (Plan-Apochromat 63x/1.40 Oil DIC M27). Signal was captured using line-scanning mode (512 pixels X 1920 lines). Custom-made IDL (interactive digital language) script was used for data analysis. Transient amplitude was expressed as $\Delta F/F_0$.

Seahorse bioanalyzer and ROS measurements

HiPSC-CMs were seeded at 30,000 per well in Matrigel-coated Seahorse plates and basal culturing conditions were performed as previously described (Guan et al., 2014). For nutrient challenge, hiPSC-CMs were cultured in RPMI-1640 B27^{+} glucose^{-} lactate^{+} three days prior to assay. HiPSC-CMs were switched to Seahorse

unbuffered RPMI-1640 supplemented with B27^{+} glucose^{-} lactate^{+} prior to the assay run. The bioenergetic responses of cells were measured with the Seahorse Bioscience XF96 flux Analyzer following instruction in the XF cell Mito Stress Test Kit User Guider. All oxygen consumption rate (OCR) measurements were acquired at 5-minute intervals with 1-minute mixing step between. Three baseline measurements were acquired followed by injection of oligomycin to a final concentration of 2.5 μM. After three measurements in presence of oligomycin, FCCP was injected to a final concentration of 1 μM and three measurements were recorded. Lastly, rotenone and antimycin A were injected to reach a final concentration of 2 μM followed by three measurements. For nutrient challenge assays, cardiomyocytes were subjected to four metabolic substrate conditions: basal assay medium (Seahorse assay medium), D-glucose (5 μM), Palmitate (167 μM), or Pyruvate (5 μM) and loaded with oligomycin (5 μM), FCCP (5 μM), rotenone and antimycin A (1.69 μM). OCR was normalized to live cell count using a PrestoBlue dye according to manufacturer's protocol. PrestoBlue (A-13261, Life Tech), Mitotracker Green (M7514, Thermo Scientific) and CellROX deep red (C10422, Thermo Scientific) were measured and quantified on a TECAN Pro1000 machine (Stanford High-Throughput Bioscience Center).

Traction Force Microscopy

Micropatterned hydrogel devices of 10 kPa and 35 kPa stiffness were manufactured 1-day prior to passaging and seeding of day 25 hiPSC-CMs. Imaging and data acquisition were consistently performed 4-5 days after seeding the cells on

the devices. HiPSC-CM derived from Isogenic pairs were always measured consecutively on the same day. HiPSC-CM derived from each cell line were measured in differentiation triplicates.

To generate micropatterned hydrogel devices, polydimethylsiloxane stamps (PDMS) (PDMS-182, Sylgard, mixed at 1:10 ratio) were prepared by replica molding of SU8-silicon master mold produced in house using photolithography (Ribeiro et al., 2015). A master mold containing several 1 cm^2 arrays of micropatterns of 1:7 (width:length) aspect ratio and 118 x 17 μm (~2000 $μm^2$) area was prepared on 4” single-side polished silicon wafer using UV lithography in 10 μm-thick SU8-2010 resin (MicroChem, USA) using a collimated light source (OAI) (130 mJ/cm^2) and a high-resolution film photomask (FineLineImaging, USA), chemical development with SU-8 developer followed by hard-bake on a 180°C hotplate for 10 min. PDMS was mixed using a Thinky^{TM} mixer, poured on the molds, degassed under vacuum for 1 h and cured in a 70°C oven for > 1 h. After carefully separating the solidified PDMS from the SU8-master mold, individual patterned stamps of were cut using a razor blade. Stamps were cleaned using a rotary shaker in a sequence of 5 min 1:1 H_2O:Ethanol followed by 5 min pure Ethanol. Stamps were subsequently dried with a gentle nitrogen stream. Stamps were cooled in a 4°C refrigerator before incubation at 4°C with 150 μl of Matrigel (Corning™ Matrigel™ hESC-Qualified Matrix, Catalog #: 08-774-552) dissolved 1:10 in Leibovitz's L-15 Medium (Thermo Fisher, Catalog #: 11415064) for a minimum of 12 hours. The stamps were then softly rinsed once with 150 μl of 4°C L-

15 medium to remove excess protein, the solution was then aspirated, and the stamps were carefully dried using a gentle nitrogen stream.

For microprinting and pattern transfer, glass cover slips of 18 mm diameter were cleaned using a rotary shaker in a sequence of 3min in Acetone followed by 3 min in Isopropanol. After drying with nitrogen, the coverslips were plasma treated for 20 s at 80 W in an oxygen plasma asher reactor (Branson IPC/Novellus). The protein patterns were then stamped onto the coverslips by carefully placing a PDMS stamp onto each freshly plasma activated glass coverslip. A glass slide was used as a carrier for the stamps and 50 g weights were placed on top of the stack for 2 min to maintain an even and constant pressure. The weights were then removed, and after 3 min the coverslips were removed from the stamps.

22mm-square coverslips were used as carrier substrates for the hydrogels and were functionalized by silanization to covalently bind the polyacrylamide gel to the glass. The coverslips were first cleaned and plasma activated, as above, and subsequently incubated with 0.3% 3-(trimethoxysilyl)propyl methacrylate (Sigma Aldrich, Catalog #: 440159) in 1:20 acetic acid glacial:ethanol (Sigma Aldrich, Catalogue #: 695092 and 459836) for 5 min. The excess solution was then aspirated, and the coverslips were rinsed using ethanol, dried with nitrogen and placed in a vacuum for a minimum of 30 min prior to usage.

Hydrogel devices were prepared by polymerization of freshly prepared and degassed polyacrylamide solution sandwiched between the transfer-substrate coverslip and the carrier substrates. Solutions of Acrylamide (Sigma Aldrich, Catalog

#: A9099) (0.5 g/ml), N,N′-Methylenebis(acrylamide) (Sigma Aldrich, Catalog # 146072) (0.025 g/ml), ammonium persulfate (APS) (Sigma) (0.1% w/v), N,N,N′,N′-tetramethylethylenediamine (TEMED)(Sigma Aldrich) (0.1% v/v), HEPES (Life Technologies) (35 mM), fluorescent microbeads (green/red fluorescent latex microsphere, diameter 0.5 µm, ThermoFischer, Catalogue #: F8813) and Milli-Q water were prepared and used throughout this study to maintain consistency. The two stiffnesses used in this study, 10 and 35 kPa, were obtained by mixing the prepolymer at 1% bisacrylamide to acrylamide ratio and 10% and 15% polymer to liquid-phase ratio, respectively. Fluorescence beads were sonicated for 10 min and pre-mixed 15% (v/v) in HEPES and added to obtain ~2% beads stock (~$6x10^9$ microbeads/mL) in the final gel solutions.

The hydrogel components were first mixed and degassed for 30 min in the absence of APS and TEMED. Immediately prior to making the gels, the APS and TEMED were added using a manual 2- and 10 µl-pipette, rapidly mixed with a 1 ml pipette, before dispensing a volume of 50 µl gel precursor on each square coverglass with a 200 µl pipette. The round coverslip containing the protein micropattern was then immediately placed on top of the gel precursor. The gel was allowed to polymerize for at least 30 min thereby transferring the micropatterns onto the solidifying hydrogel. Once polymerization was complete, the hydrogel devices were transferred into 6-well cell-culture dishes and incubated at room temperature overnight in Phosphate Buffer Saline (PBS) with 1% PenStrep to allow for swelling of the hydrogels. 15 min prior to seeding hiPSC-CMs, the top round coverglass used to transfer the protein

micropatterns was removed from the top of the hydrogel surface to expose the protein micropatterns using precision forceps. The PBS solution was aspirated, and the devices were pre-conditioned in a cell culture incubator at 37°C until seeding of the cells.

HiPSC-CMs were passaged onto micropatterned polyacrylamide devices by using 4:1 Accutase:TrypLE™ for dissociation and RPMI-1640 B27$^+$ glucose$^-$ lactate$^+$ medium with 5 μM ROCK inhibitor and 10% Knock-out Serum (KSR) for resuspension. After counting, cells were deposited onto the 18 mm diameter hydrogels at a density of ~30,000 cells/cm^2 in a final 150 µl volume at a concentration of 200,000 cell/ml. After 30 min incubation, additional 2 ml of medium was added. After overnight incubation, the medium was replaced with fresh RPMI-1640 B27$^+$ glucose$^-$ lactate$^+$ medium and replaced every 2 days thereafter.

Supplemental References

Guan, X., Mack, D.L., Moreno, C.M., Strande, J.L., Mathieu, J., Shi, Y., Markert, C.D., Wang, Z., Liu, G., Lawlor, M.W., Moorefield, E.C., Jones, T.N., Fugate, J.A., Furth, M.E., Murry, C.E., Ruohola-Baker, H., Zhang, Y., Santana, L.F., Childers, M.K., 2014. Dystrophin-deficient cardiomyocytes derived from human urine: new biologic reagents for drug discovery. Stem Cell Res 12, 467–480. doi:10.1016/j.scr.2013.12.004

Wu, H., Yang, H., Rhee, J.-W., Zhang, J.Z., Lam, C.K., Sallam, K., Chang, A.C.Y., Ma, N., Lee, J., Zhang, H., Blau, H.M., Bers, D.M., Wu, J.C., 2019. Modelling diastolic dysfunction in induced pluripotent stem cell-derived cardiomyocytes from hypertrophic cardiomyopathy patients. Eur. Heart J. 40, 3685–3695. doi:10.1093/eurheartj/ehz326

Supplemental Table S1. Summary of Control and DMD hiPSC lines used.

**Patient hiPSC lines**						
**Classification**	**hiPSC lines**	**Mutation**	**Age**	**Tissue**	**Gender**	**Source**
Healthy	Con#1	Isogenic control of DMD #1	10	Skin	male	University of Nottingham
Healthy	Con#2	Isogenic control of DMD #2	8	Skin	male	University of Nottingham
Healthy	Con#3	Isogenic control of DMD #3	16	Urine mesenchymal	male	University of Washington
Healthy	Con#4	Healthy control	45	PBMC	male	Stanford
Healthy	Con#5	Healthy control	62	T-Cells	male	Harvard
Healthy	Con#6	Healthy control	48	T-Cells	female	Harvard
DMD	DMD#1	DMD (c.10171C>T)	10	Skin	male	University of Nottingham
DMD	DMD#2	DMD (c.4918_4919delACinsTG)	8	Skin	male	University of Nottingham
DMD	DMD#3	DMD (c.19delG)	16	Urine mesenchymal	male	University of Washington
DMD	DMD#4	DMD (c.3638_3650del)	6	PBMC	male	Stanford
DMD	DMD#5	DMD (c.6599 C>G)	12	PBMC	male	Stanford
DMD	DMD#6	DMD (c.9204_9207del)	9	PBMC	male	Stanford

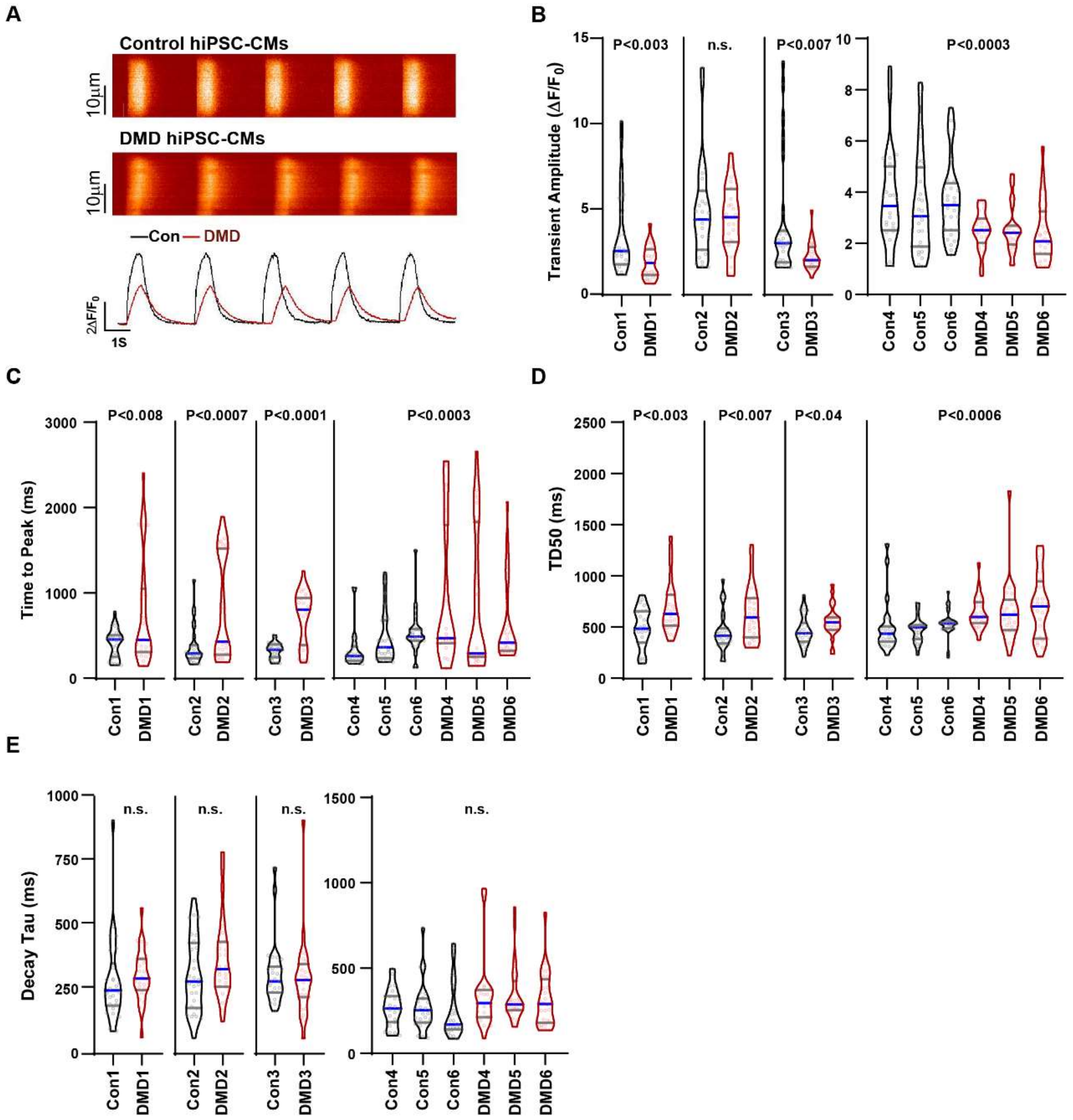

Figure S1. Evaluation of spontaneous calcium handling in control and DMD hiPSC-CMs. (**A**) Representative fluorescent based Fluo-4 calcium imaging recording traces in Con and DMD iPSC-CMs, (**B**) transient amplitude, (**C**) time to peak, (**D**) TD50, and (**E**) decay tau are plotted (n = 3 independent experiments, 24-28 cells analyzed). Data are shown as violin plots where blue median and gray quartiles are shown. Student's t-test used for Con/DMD 1-3. One-way ANOVA with Holm-Sidak's multiple comparison test used for Con/DMD 4-6.

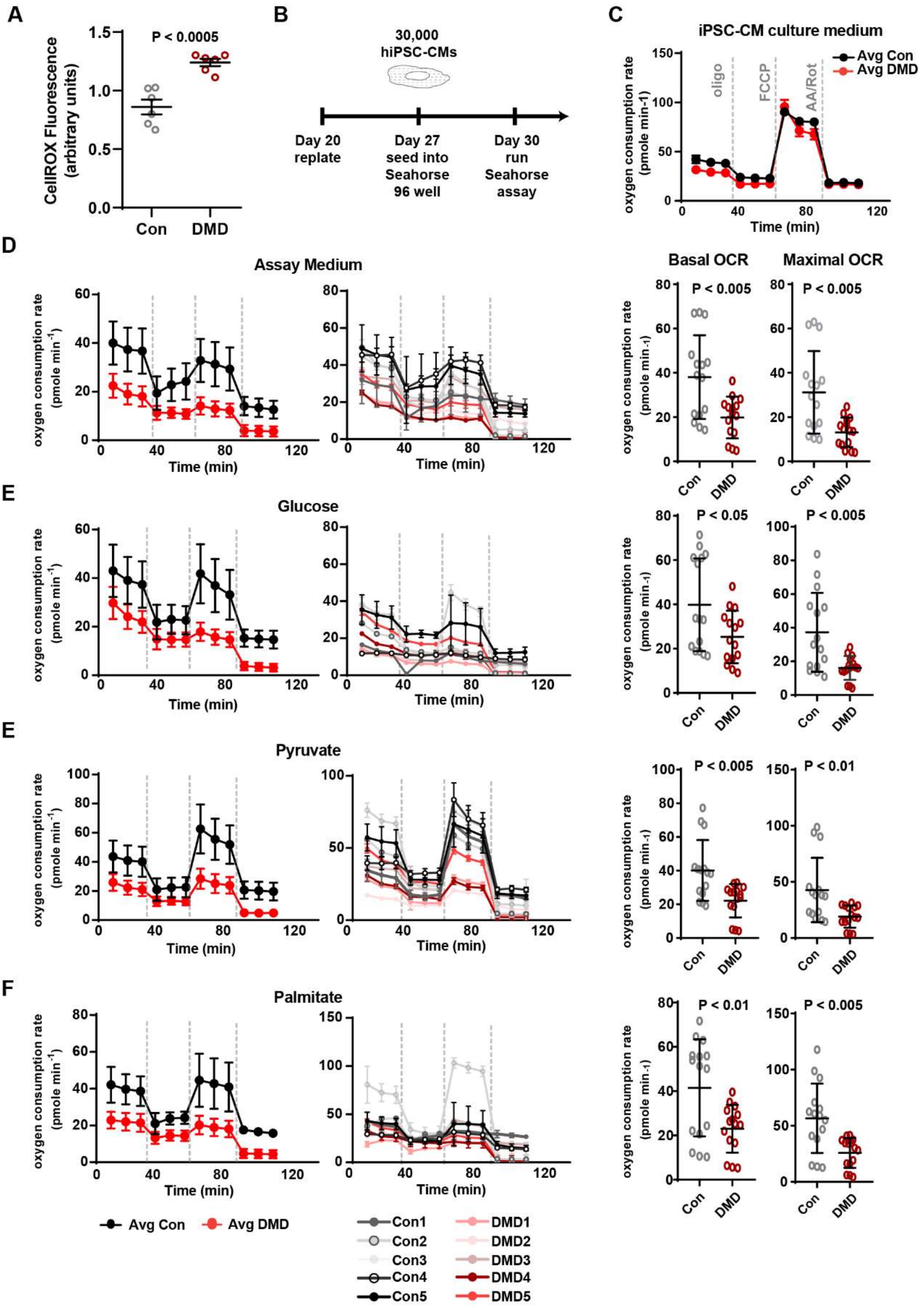

Figure S2. **DMD hiPSC-CMs exhibit mitochondrial dysfunction when subjected to nutrient challenge.** (**A**) Intracellular reactive oxygen species were measured in Con and DMD hiPSC-CMs (n = 6 independent experiments). (**B**) Real-time oxygen

consumption rate (OCR) were evaluated for control and DMD hiPSC-CMs under (**C**) normal culture conditions, (**D**) assay medium only, (**E**) glucose only, (**F**) pyruvate only, and (**G**) palmitate only conditions (n = 5 independent experiments). Data are represented as mean ± SEM. Student's t-test used for statistical analysis.

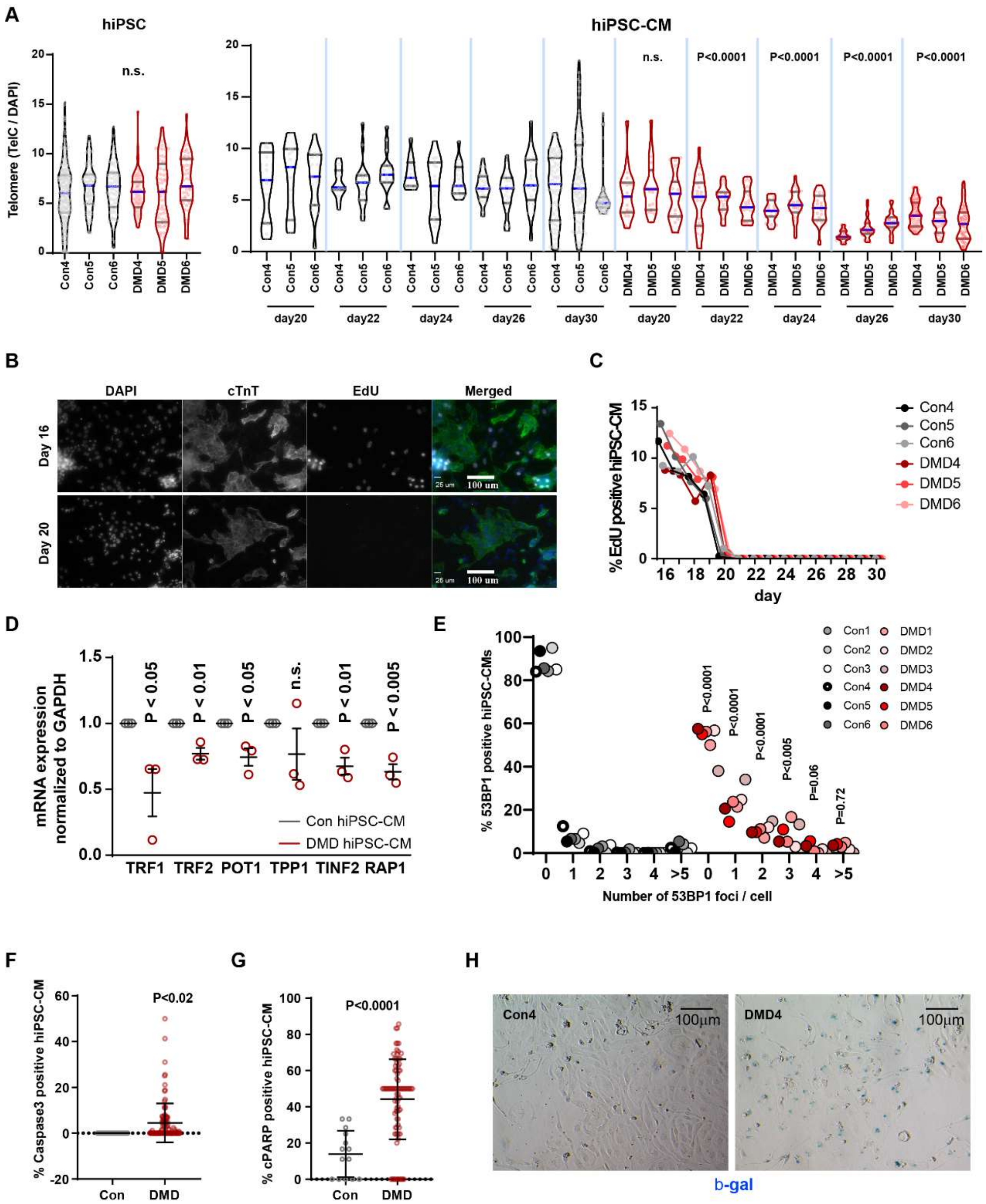

Supplemental Figure S3. (**A**)Quantification of hiPSC (n = 56-175 independent experiments) and hiPSC-CMs (n = 3 independent experiments, 13-108 cells analyzed) between days 20 to 30 of Con/DMD 4-6 lines. Data are shown as violin plots where blue median and gray quartiles are shown. One-way ANOVA with Holm-Sidak's multiple comparison test used for Con/DMD 4-6. (**B**) HiPSC-CM proliferation status evaluated by microscopy of EdU staining (24 hr pulses) and (**C**)

quantified for Con/DMD 4-6 lines. (**D**) Endogenous shelterin expression levels were determined by RT-qPCR in TMRM-purified day 20 hiPSC-CMs (n = 3 independent experiments). (**E**) Quantification of 53BP1 foci by immunofluorescence staining for Con and DMD hiPSC-CMs (n = 6 independent experiments). Data represent as mean ± SEM. Student's t-test used for statistical analysis. Quantification of (**F**) caspase 3 (n = 3 independent experiments, 23-101 cells analyzed) and (**G**) cleaved PARP (n = 3 independent experiments, 14-88 cells analyzed) by immunofluorescence staining for Con and DMD hiPSC-CMs. Data represent as mean ± SEM. Student's t-test used for statistical analysis. (**H**) Representative brightfield image of Control and DMD hiPSC-CMs stained for β-galactosidase.

Supplemental Videos:

Video S1. Brightfield of Control iPSC-CM on biopatterned hydrogel.

Video S2. Fluorescent channel of Control iPSC-CM on biopatterned hydrogel.

Video S3. Traction heatmap of Control iPSC-CM on biopatterned hydrogel.

Video S4. Brightfield of DMD iPSC-CM on biopatterned hydrogel.

Video S5. Fluorescent channel of DMD iPSC-CM on biopatterned hydrogel.

Video S6. Traction heatmap of DMD iPSC-CM on biopatterned hydrogel.
